# Hsp90 Promotes Gastric Cancer Cell Metastasis and Stemness by Regulating the Regional Distribution of Glycolysis‐Related Metabolic Enzymes in the Cytoplasm

**DOI:** 10.1002/advs.202310109

**Published:** 2024-06-14

**Authors:** Shiya Liu, Gaigai Shen, Xuanyu Zhou, Lixin Sun, Long Yu, Yuanting Cao, Xiong Shu, Yuliang Ran

**Affiliations:** ^1^ State Key Laboratory of Molecular Oncology National Cancer Center/National Clinical Research Center for Cancer/Cancer Hospital Chinese Academy of Medical Sciences and Peking Union Medical College Beijing 100021 China; ^2^ Department of Epidemiology & Population Health Stanford University of Medicine Stanford CA 94305 USA; ^3^ Beijing Research Institute of Orthopaedics and Traumatology Beijing Jishuitan Hospital Capital Medical University Beijing 100035 China

**Keywords:** combination therapy, glycolysis, Hsp90, multienzyme complex, regionalized distribution

## Abstract

Heat‐shock protein 90 (Hsp90) plays a crucial role in tumorigenesis and tumor progression; however, its mechanism of action in gastric cancer (GC) remains unclear. Here, the role of Hsp90 in GC metabolism is the focus of this research. High expression of Hsp90 in GC tissues can interact with glycolysis, collectively affecting prognosis in clinical samples. Both in vitro and in vivo experiments demonstrate that Hsp90 is able to regulate the migration and stemness properties of GC cells. Metabolic phenotype analyses indicate that Hsp90 influences glycolytic metabolism. Mechanistically, Hsp90 interacts with glycolysis‐related enzymes, forming multienzyme complexes to enhance glycolysis efficiency and yield. Additionally, Hsp90 binds to cytoskeleton‐related proteins, regulating the regional distribution of glycolytic enzymes at the cell margin and lamellar pseudopods. This effect could lead to a local increase in efficient energy supply from glycolysis, further promoting epithelial‐mesenchymal transition (EMT) and metastasis. In summary, Hsp90, through its interaction with metabolic enzymes related to glycolysis, forms multi‐enzyme complexes and regulates regional distribution of glycolysis by dynamic cytoskeletal adjustments, thereby promoting the migration and stemness of GC cells. These conclusions also support the potential for a combined targeted approach involving Hsp90, glycolysis, and the cytoskeleton in clinical therapy.

## Graphical abstract

Heat‐shock protein 90 (Hsp90) participates in regulating tumor progression by modulating protein stability. This study reveals that Hsp90 interacts with glycolytic enzymes to form multi‐enzyme complexes and regulates the intracellular regional distribution of glycolysis through cytoskeletal dynamics, thereby promoting the metastasis and stemness of gastric cancer cells. It also highlights the potential of Hsp90 and glycolysis as combined therapeutic targets.

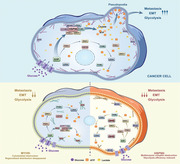

## 1 Introduction

Globally, gastric cancer (GC) occupies the fifth position in terms of incidence and fourth position in terms of mortality.^[^ ^1^ ^]^ Although its incidence has decreased at the global level,^[^ ^2^ ^]^ China and other East Asian regions still have the world's highest incidence of GC.^[^ ^3^ ^]^ Despite significant advances in surgery, radiotherapy, chemotherapy, and immunotherapy, the overall survival rate of patients with GC remains relatively poor due to the complex nature of the tumor's driving genetic factors, high intra‐tumoral and inter‐tumoral heterogeneity, and the presence of tumor stem cells. GC is prone to recurrence and metastasis,^[^ ^4^ ^]^ and there is still a lack of effective targeted therapies.^[^ ^5^ ^]^ Extensive research is needed to uncover the molecular mechanisms of potential targets in GC and identify novel therapeutic strategies to mitigate the disease. The level of glycolysis in tumor cells is significantly enhanced, and the cells favor the glycolytic pathway for energy production not only under hypoxic but also under aerobic conditions.^[^ ^6^ ^]^ As a result of research into the Warburg effect, there has been increasing in vivo evidence for the occurrence of this metabolic reprogramming phenomenon in many tumor types. It is closely associated with the maintenance of the tumor stem cell state, cancer progression and migration, and drug resistance.^[^ ^7^ ^]^ Metabolism, primarily glycolysis, is highly intertwined with the phenomena of epithelial‐mesenchymal transition (EMT) and acquisition of stem‐like properties within tumor tissues.^[^ ^8^ ^]^ We previously reported that changes in glycolysis levels in tumor cells were associated with stem‐like characteristics displayed by the entire tumor cell population.^[^ ^9^ ^]^ However, due to the existence of tumor heterogeneity, the specific regulatory mechanisms remained unclear. Some studies have proposed that glycolysis influences tumor progression by altering the protein levels of glucose metabolism transporters and enzymes.^[^ ^10^ ^]^ Limited reports are available on the regulatory mechanisms of glycolytic enzyme activity. Preliminary investigations have suggested that actin's potential role in modulating aldolase A release and boosting its activity.^[^ ^11^ ^]^ Hence, it becomes imperative to decipher the regulation of glycolysis in tumor cells and understand how it precisely controls the occurrence of EMT and metastasis in tumors. Such explorations hold notable clinical significance for identifying specific targets and inhibitors in the metabolism and stem‐like properties of tumor cancer stem cells (CSCs).^[^ ^12^ ^]^

Heat‐shock protein 90 (Hsp90) is a highly conserved group of molecular proteins in the heat‐shock protein family, which interact with client proteins and co‐chaperones to function. Hsp90 plays a significant role in vital physiological processes such as apoptosis, protein folding and degradation, and other fundamental cellular processes and regulatory pathways.^[^ ^13^ ^]^ Hsp90 is widely expressed in most tumors. It can regulate the late‐stage maturation, activation, and stability of various client proteins that participate in the processes of tumor growth, invasion, and metastasis.^[^ ^14^ ^]^ It is also associated with adverse clinical pathological features and poor prognosis in various tumors such as GC, breast cancer, liver cancer, and others.^[^ ^15^ ^]^ In various tumors, including liver cancer and prostate cancer, Hsp90 has been linked to abnormally activated glycolysis in tumor cells.^[^ ^16^ ^]^ However, in GC, there have been no relevant reports as to whether Hsp90 can regulate the occurrence of EMT in tumor cells through glycolysis promotion, and the specific molecular mechanisms involved remain obscure.

Inhibitors targeting Hsp90 and glycolysis have been applied in clinical settings.^[^ ^17^ ^]^ However, their inevitable off‐target effects may lead to noticeable hepatotoxicity and ocular toxicity.^[^ ^18^ ^]^ This limitation has hindered the clinical application of such drugs. Thus, it is crucial to optimize therapeutic effects while minimizing drug toxicity, especially for the treatment of GC. The exploration of effective and low‐toxicity combination therapies poses a significant challenge to the clinical drug strategy for GC.

Our study revealed that Hsp90 plays a crucial role in regulating the stemness properties and malignant phenotype of tumor cells. In particular, we focused on how Hsp90 affects the reprogramming of glucose metabolism in tumor cells and the molecular mechanism underlying this phenomenon, with the aim of uncovering a strategic approach to the treatment of GC using a combination of drugs.

## 2 Results

### 2.1 Upregulation of Hsp90 in GC May Potentially Synergize with Glycolysis to Affect Clinical Prognosis

**Figure 1:**
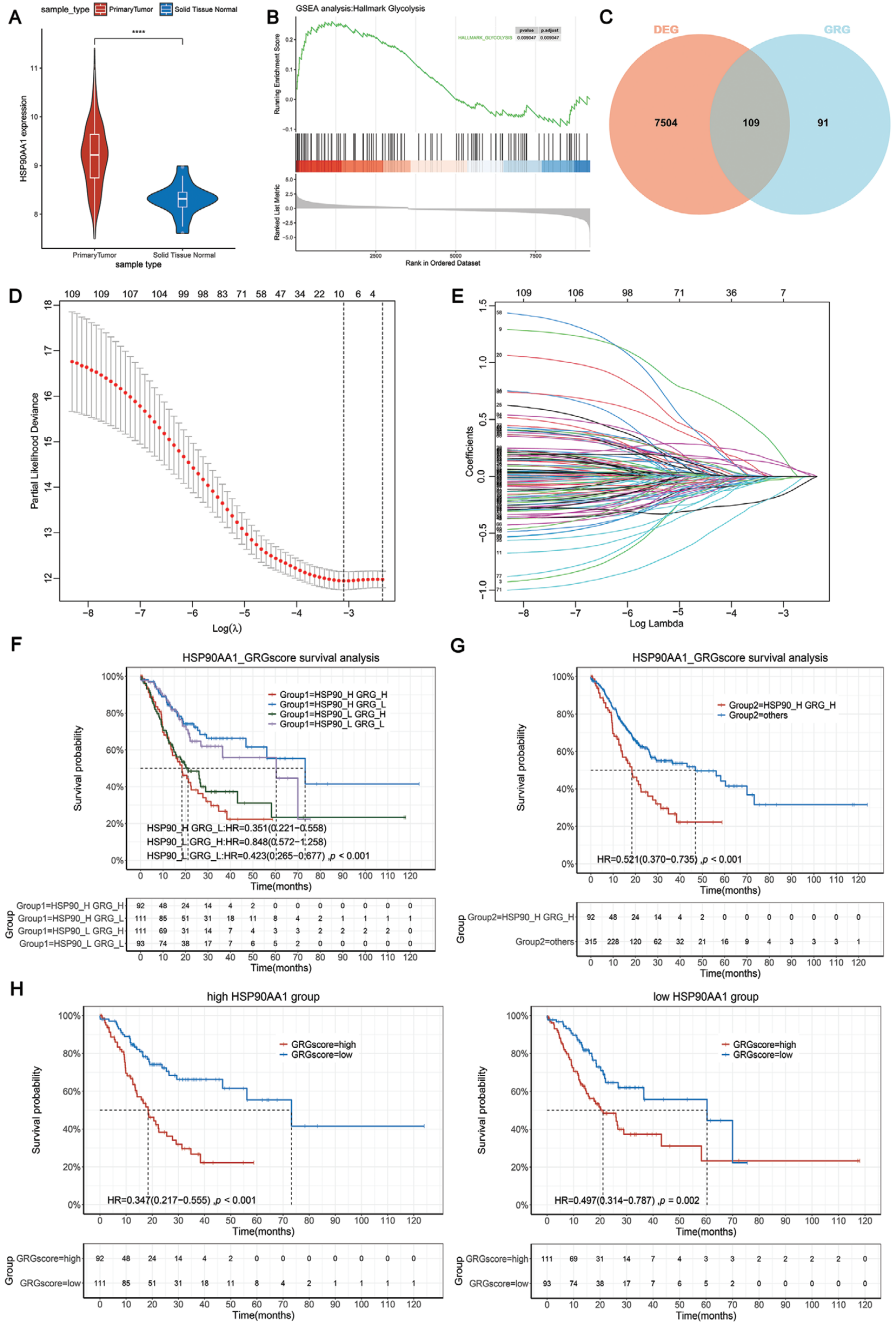
Upregulation of Hsp90 in gastric cancer (GC) may potentially synergize with glycolysis to affect clinical prognosis. A) The expression of Hsp90 in patients with GC was derived from TCGA database. B) GSEA analysis was performed on HALLMARK_GLYCOLYSIS gene sets in gastric cancer. C) Venn diagram shows the intersection of glycolytic‐related genes with GC differential genes in the TCGA dataset. D,E) Lasso regression of glycolysis‐related genes (GEGs) and cross‐validation to determine the optimal penalty parameter λ. F,G) Prognostic analysis of GC patients was performed in combination with glycolysis and Hsp90. The KM plots show overall survival in F) 4 groups and G) 2 newly defined groups. H) Prognostic analysis was performed for glycolysis scores in GC patients with high and low Hsp90 expression.

To explore the interaction between Hsp90 and glycolysis in tumors, we utilized relevant data from The Cancer Genome Atlas (TCGA) database, and found that Hsp90 was highly expressed at mRNA level in GC patients (**Figure** **1**A). After removing duplicates and missing values, 407 tumor samples and 36 normal samples were included for follow‐up analysis. Then, we downloaded the glycolysis gene set (HALLMARK_GLYCOLYSIS) and found that the glycolysis genes were significantly enriched in the patients with GC (Figure 1B). Combined with differentially expressed genes in tumors, ultimately, 109 glycolytic genes were selected as research targets that were differentially expressed in tumor tissues (Figure 1C). The 109 genes were analyzed with LASSO regression analysis (Figure 1D,E). Lastly, a GRG model consisting of six mRNAs (AURKA, VLDLR, P4HA1, EFNA3, PKP2, TALDO1) was developed. The glycolysis score was calculated according to the following formula: GRG score = (0.79524227 × AURKA expression) + (−0.1595481 × VLDLR expression) + (0.23009793 × P4HA1 expression) + (−0.3490827 × EFNA3 expression) + (−0.4610763 × PKP2 expression) + (0.31310546 × TALDO1 expression). STAD patients were divided into the high‐glycolysis group and the low‐glycolysis group according to their GRG score. Subsequently, STAD patients were classified into four molecular subtype groups based on their Hsp90 expression and GRG score and categorized according to the median, namely as Hsp90 High + GRG score High, Hsp90 High + GRG score Low, Hsp90 Low + GRG score High, and Hsp90 Low + GRG score Low. Prognostic analysis results indicated that the patients in the Hsp90 High + GRG score High group exhibited a shorter survival time than those in the other three groups (Figure 1F). Additionally, prognostic analysis of Hsp90 High + GRG score High group, when compared to other STAD patients, revealed a *p*‐value <0.001 (Figure 1G). To further explore the cross‐linking mechanism, we analyzed the Hsp90 high‐expression and the Hsp90 low‐expression groups, and found that the hazard ratio (HR) value of the prognostic analysis within the high and low glycolysis groups was significantly lower than that of the low Hsp90 expression group. This indicates that the high expression of Hsp90 makes glycolysis more influential on the prognosis of patients (Figure 1H). These results suggest that high Hsp90 levels and high glycolysis are associated with an unfavorable prognosis.

### 2.2 High Hsp90 Promotes Proliferation, Metastasis, and Other Stem‐Related Features of GC In Vivo and In Vitro

**Figure 2:**
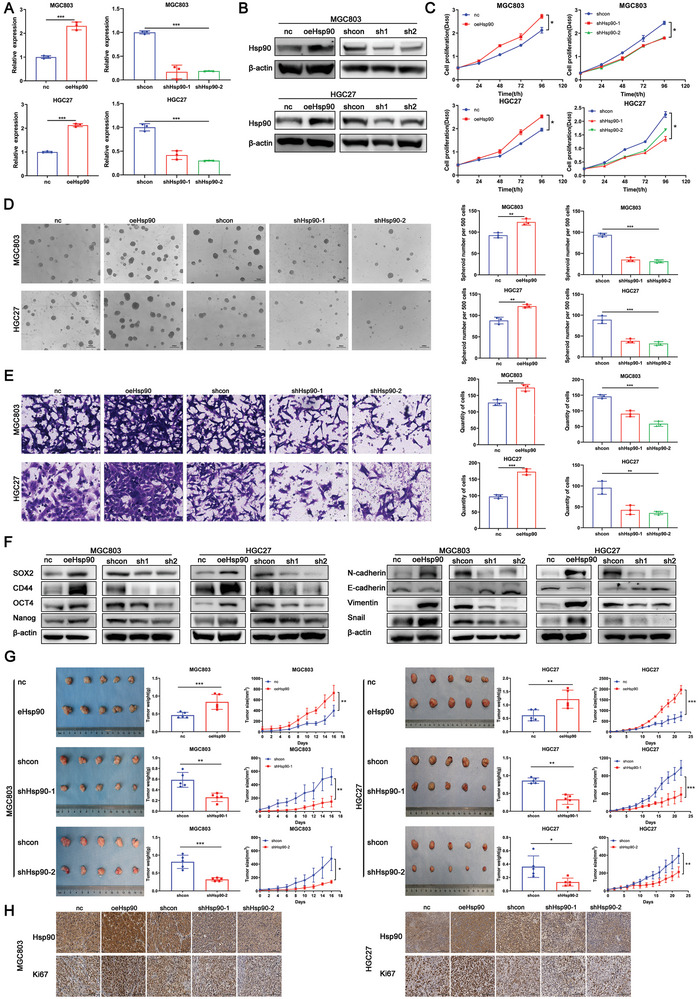
High Hsp90 promotes proliferation, metastasis and other stem‐related features of gastric cancer in vivo and in vitro. A,B) Quantitative RT‐PCR and western blot for Hsp90 expression indicated in GC transfected cell lines with stable Hsp90 knockdown and overexpression. C) CCK‐8 show cell proliferation capacity of transfected cells. D) Analysis of the self‐renewal abilities of MGC803 and HGC27 cells stably expressing nc, oeHsp90, shcon, or shHsp90. Scale bar, 1000 µm. E) Analysis of the invasion abilities of MGC803 and HGC27 cells stably expressing nc, oeHsp90, shcon, or shHsp90. Scale bar, 100 µm. F) Expression of stemness markers and EMT‐related markers in MGC803 and HGC27 stable cell lines was detected by western blot. G) Tumorigenicity was evaluated in MGC803 and HGC27 cells stably expressing nc, oeHsp90, shcon, or shHsp90 (*n* = 5). H) IHC for Hsp90 and Ki67 in serial sections of tumor tissues from mice injected with MGC803 and HGC27 cells stably expressing nc, oeHsp90, shcon, or shHsp90. Scale bar, 100 µm. Error bars indicate mean ± SD. *p < 0.05, **p < 0.01 and ***p < 0.001.

To investigate the effect of Hsp90 on stem cell‐like features of GC, we first used lentiviral transfection techniques to continuously knock down and overexpress Hsp90 in MGC803 and HGC27 cells, which was further confirmed by PCR and WB (**Figure** **2**A,B). Next, we investigated the role of Hsp90 in stem‐like properties using the stably transfected cell lines. Initially, we analyzed the influence of Hsp90 on GC cell proliferation and colony‐formation ability. Overexpression of Hsp90 (oeHsp90) significantly increased MGC803 and HGC27 cell proliferation and colony formation (Figure 2C and Figure S1A, Supporting Information). In contrast, compared with the control cells, Hsp90‐knockdown (shHsp90) cells showed significantly reduced proliferation and colony‐forming capacity. In sphere‐formation experiments, we observed a significant increase in the self‐renewal ability of oeHsp90 cells. In addition, the self‐renewal capacity of shHsp90 cells was significantly reduced compared to the control cells (Figure 2D).

High metastatic ability and tumorigenicity are potentially crucial attributes of stem‐like cancer cells. To demonstrate the influence of Hsp90 on the migration and invasion capabilities of tumor cells, we conducted experiments on migration and invasion. The oeHsp90 cells had higher migration and invasion rates than cells in the control group (oeNC and shcon), whereas shHsp90 cells had significantly lower migration and invasion rates (Figure S1B, Supporting Information, Figure 2E). We also employed the plate‐scratch assay for further verification (Figure S1C, Supporting Information). We further verified our observations of stemness by analyzing the changes in stemness‐related molecular markers (including CD44, Nanog, Oct4, and Sox2) and EMT‐related markers (including N‐cadherin, E‐cadherin, vimentin, and snail). The markers were significantly increased in oeHsp90 cells but significantly decreased in shHsp90 cells (Figure 2F and Figure S1D, Supporting Information). Flow cytometry results for oeHsp90 GC cells revealed a substantial increase in the percentages of CD90^+^ proliferative and CD44^+^ metastatic stem cells, whereas such percentages decreased in the knockdown group (Figure S1E, Supporting Information). Then, we subcutaneously injected oeHsp90, shHsp90, and the corresponding control cells (oeNC, shcon) into nude mice. Our findings revealed that tumors derived from oeHsp90 cells grew faster and weighed more than those from corresponding control cells. On the other hand, tumors from shHsp90 cells grew slower and weighed much less (Figure 2G). In addition, IHC further verified the expression of Hsp90 in the tumors, and Ki67 was used to further verify the proliferation of the tumor tissues (Figure 2H). These results indicate that oeHsp90 cells have a stronger tumorigenic potential, while shHsp90 cells have a weaker tumorigenic potential. In conclusion, we demonstrated that Hsp90 can enhance the stem‐like characteristics of GC cells in terms of functional phenotype and molecular phenotype.

### 2.3 Hsp90 Can Interact with Metabolic Enzymes Associated with Glycolysis and Positively Regulates Glycolysis Levels in GC Cells

**Figure 3:**
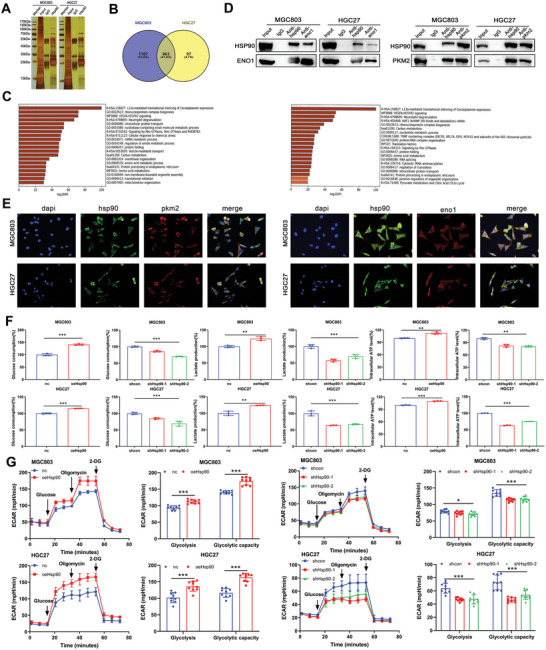
Hsp90 can interact with metabolic enzymes associated with glycolysis and positively regulates glycolysis levels in GC cells. A) Cellular lysates from MGC803 and HGC27 cells were separated and purified by antibodies and magnetic beads. The eluates were separated by SDS‐PAGE and stained with silver. B) Preliminary analysis of mass spectrometry results and the pull‐down proteins of the two cell lines were shown by Venn diagrams. C) The differential protein bands were retrieved and analyzed by mass spectrometry. Analysis of proteomic data showed that it could be enriched to carbon metabolism. D) MGC803 and HGC27 cells were immunoprecipitated with normal IgG, anti‐Hsp90 or other antibody, and precipitates were analyzed by immunoblotting (IB) with indicated antibodies. E) The colocalization of Hsp90, ENO1 and PKM2 in MGC803 and HGC27 cells was demonstrated by immunofluorescence. Scale bar, 100 µm. F) Glucose consumption, lactate production and intracellular ATP production in MGC803 and HGC27 cells stably expressing nc, oeHsp90, shcon, or shHsp90. G) ECAR were examined in MGC803 and HGC27 cells stably expressing nc, oeHsp90, shcon, or shHsp90. Error bars indicate mean ± SD. *p < 0.05, **p < 0.01 and ***p < 0.001.

To better explore the molecular mechanism of Hsp90 in GC cells, we initially attempted to identify the proteins that directly interact with Hsp90 in GC cells using IP and to identify the purified proteins by mass spectrometry (MS) analysis. MS data indicated that Hsp90 may be associated with ENO1 and PKM2 (**Figures** **3**A–C and S2, Supporting Information). To confirm potential interactions between Hsp90 and some glycolytic metabolic enzymes identified by LCMS, we performed Co‐IP analysis with Hsp90 antibodies. The expression of ENO1 and PKM2 was detected in cell lysates after immunoprecipitation with Hsp90‐coupled magnetic beads. Hsp90 coprecipitated with ENO1 and PKM2, indicating that endogenous Hsp90 could interact with ENO1 and PKM2 (Figure 3D). Immunofluorescence staining showed that Hsp90 colocalized with ENO1 and PKM2 in the membrane and cytoplasm of both cell lines (Figure 3E). We further demonstrated that Hsp90 can interact with glycolysis‐related metabolic enzymes.

Next, we investigated whether Hsp90 regulates stem‐like characteristics such as proliferation, migration, and invasion by affecting glycolysis metabolic enzymes. We measured changes in glycolysis levels in overexpressed and knockdown cells with regard to the corresponding control cells. The results revealed that glucose consumption, lactic acid production, and ATP production were increased in oeHsp90 cells. In contrast, after stable knockdown of Hsp90, the glucose consumption, lactic acid production, and ATP production were significantly reduced (Figure 3F). To further validate Hsp90's effect on glycolysis, we measured extracellular acidification rate (ECAR) in these stable cell lines. Consistent with our hypothesis, overexpression of Hsp90 increased ECAR, whereas decreased ECAR was observed in shHsp90 cells (Figure 3G). This suggests that Hsp90 can promote glycolysis in GC cells.

### 2.4 Glycolysis Can Affect the Stem‐Like Characteristics of GC Cells, and the Regulation of Hsp90 on Tumors Depends on Glycolysis

To confirm whether glycolysis can inhibit the stem‐like characteristics of these cells, we treated MGC803 and HGC27 cells with the glycolysis inhibitor 2‐DG. The results showed that treatment with 2‐DG (10 × 10^−3^ m or 20 × 10^−3^ m) significantly inhibited glycolysis levels and ECAR (Figure S3A,B, Supporting Information). Then, we evaluated the changes in the functional phenotype of GC cells after 2‐DG treatment. The results showed that 2‐DG treatment reduced the migration and invasion ability, self‐renewal ability, and other stem‐like characteristics of the two cell lines (Figure S3C–F, Supporting Information). Furthermore, 2‐DG treatment significantly inhibited the expression of stemness markers and EMT‐related markers (Figure S3G, Supporting Information). These results suggest that the inhibition of glycolysis is associated with the stemness of GCs.

**Figure 4:**
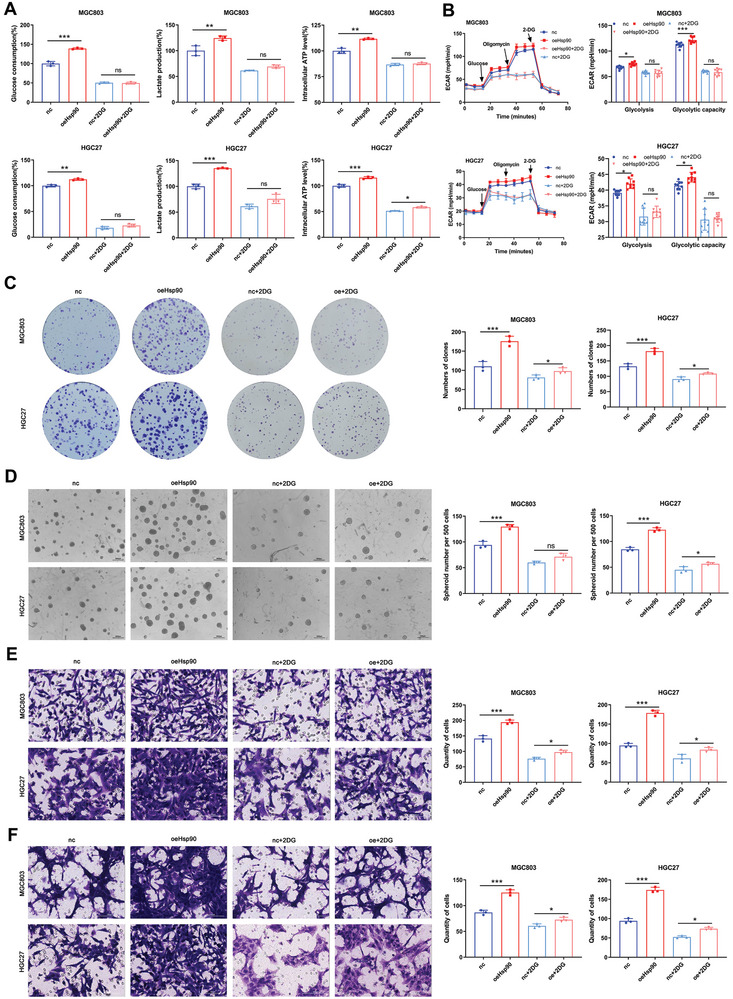
The regulation of Hsp90 on gastric cancer depends on glycolysis. A) Glucose consumption, lactate production and intracellular ATP production in MGC803 and HGC27 stable cell lines were detected before and after 2‐DG inhibition of glycolysis. B) ECAR were examined in MGC803 and HGC27 stable cell lines were detected before and after 2‐DG inhibition of glycolysis. C) The colony‐formation ability of stable cell lines was detected before and after 2‐DG inhibition of glycolysis. D) The self‐renewal ability of stable cell lines was detected before and after 2‐DG inhibition of glycolysis. Scale bar, 1000 µm. E) The migration ability of stable cell lines was detected before and after 2‐DG inhibition of glycolysis. F) The invasion ability of stable cell lines was detected before and after 2‐DG inhibition of glycolysis. Scale bar, 100 µm. Error bars indicate mean ± SD. *p < 0.05, **p < 0.01 and ***p < 0.001.

To determine the mechanism by which Hsp90 affects the stem‐like characteristics of GC cells through glycolysis, we treated MGC803 and HGC27 cells stably overexpressed and knockdown Hsp90 with 2‐DG and detected the changes in their glycolysis levels. The data indicated that 2‐DG treatment significantly restored the glycolysis levels (including glucose consumption, lactic acid production, and ATP production) and ECAR, which were increased by Hsp90 overexpression (**Figure** **4**A,B). Simultaneously, 2‐DG treatment significantly decreased the initial contrast in glycolysis levels between the control and knockdown groups (Figure S4A,B, Supporting Information). 2‐DG treatment significantly restored self‐renewal and proliferation abilities that were enhanced by Hsp90 overexpression (Figure 4C,D). It also significantly reduced the original differences in self‐renewal and proliferation ability between the control and knockdown groups (Figure S4C,D, Supporting Information). We then evaluated the effect of the same treatment on the performance of stable cell lines, and the results showed that 2‐DG treatment significantly restored the enhanced migration and invasion ability caused by Hsp90 overexpression (Figure 4E,F). Inhibiting the glycolysis level of both the Hsp90‐knockdown group and the control group also significantly reduced the initial difference in migration and invasion ability between the control group and the knockdown group (Figure S4E,F, Supporting Information). Hence, 2‐DG can recover the changes in glycolysis level and stem‐like characteristics of GC cells caused by the change in Hsp90 expression level, suggesting that the effect of Hsp90 on the stemness of tumor cells depends on glycolysis.

### 2.5 Hsp90 Forms Protein Complexes with Glycolysis‐Related Metabolic Enzymes to Improve Glycolytic Efficiency and Regulate the Regionalized Distribution of Glycolytic Enzyme Complexes by Binding to Cytoskeleton‐Related Proteins

**Figure 5:**
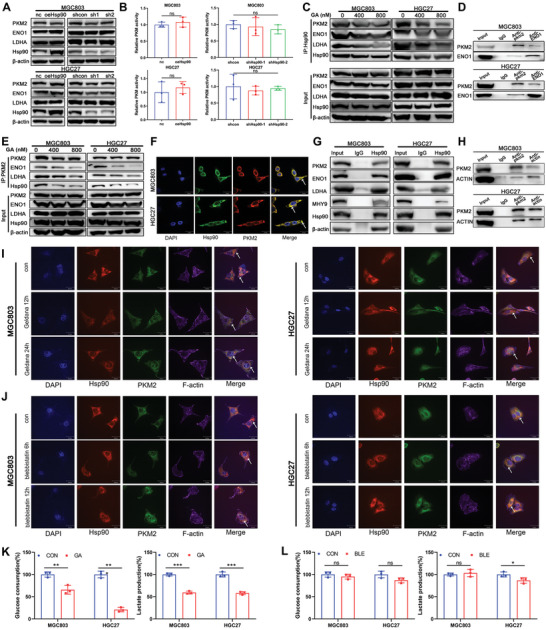
Hsp90 forms protein complexes with glycolytic‐related metabolic enzymes to improve glycolytic efficiency and regulate the regionalized distribution of glycolytic enzyme complexes. A) Expression of glycolytic enzymes in MGC803 and HGC27 stable cell lines was detected by western blot. B) Detection of PKM2 enzyme activity in MGC803 and HGC27 stable cell lines. C) Detection of protein expression of glycolytic enzyme and Hsp90 interacting glycolytic enzyme after treatment with Hsp90 inhibitor. D) The existence of glycolysis‐related enzyme complex was demonstrated by Co‐IP. E) Detection of protein expression of glycolytic enzyme and PKM2 interacting glycolytic enzyme after treatment with Hsp90 inhibitor. F) The colocalization of Hsp90 and PKM2 in MGC803 and HGC27 cells was demonstrated by immunofluorescence. Scale bar, 30 µm. G) MGC803 and HGC27 cells were immunoprecipitated with normal IgG or anti‐Hsp90, and precipitates were analyzed by immunoblotting (IB) with indicated antibodies. H) The interaction between glycolytic enzymes and cytoskeleton were demonstrated by Co‐IP. I) The changes of co‐localization positions of Hsp90 and PKM2 were detected by immunofluorescence after treatment with Hsp90 inhibitor geldanamycin (GA) for 12 h and 24 h. Scale bar, 30 µm. J) The changes of co‐localization positions of Hsp90 and PKM2 were detected by immunofluorescence after treatment with MYH9 inhibitor blebbistatin for 6 h and 12 h. Scale bar, 30 µm. K) Glucose consumption and lactate production in MGC803 and HGC27 cell were detected after GA inhibition of Hsp90. L) Glucose consumption and lactate production in MGC803 and HGC27 cell were detected after blebbistatin inhibition of MYH9. Error bars indicate mean ± SD. *p < 0.05, **p < 0.01 and ***p < 0.001.

To further explore the relationship between Hsp90 and glycolytic metabolic enzymes in GC cells, we measured PKM2 and ENO1 protein levels from stable cell lines differentially expressing Hsp90. The results indicated that PKM2 and ENO1 mRNA levels and protein levels did not change significantly (**Figures** **5**A and S5A, Supporting Information), suggesting that Hsp90 does not affect glycolysis levels by affecting the production of glycolytic metabolic enzymes. Further examination of PKM2 enzyme activity revealed that there was no modification in metabolic enzyme activity after the differential expression of Hsp90 (Figure 5B). Subsequently, we discovered that the utilization of Hsp90 inhibitors did not affect the content of glycolytic enzymes, but it did reduce the quantity of proteins bind to Hsp90 (Figure 5C). Co‐IP revealed interactions between glycolytic enzymes to demonstrate the existence of glycolysis‐related enzyme complex (Figure 5D). Further IP results showed that treatment with Hsp90 inhibitors reduced the content of glycolytic enzymes bound to PKM2, suggesting that the formation of glycolytic enzyme complexes is dependent on HSP90 (Figure 5E). Meanwhile, closer examination of Hsp90 colocalization with ENO1 and PKM2 using high‐power microscopy revealed that the colocalization was mainly concentrated at the cellular periphery (Figure 5F and Figure S5B, Supporting Information). Additionally, MS demonstrated that the Hsp90‐interacting proteins mainly included MYH9 and actin, which are significant constituents of the cytoskeleton (Figure S5C, Supporting Information). IP validation confirmed the binding of Hsp90 to glycolytic enzymes and cytoskeleton‐associated proteins (Figure 5G and Figure S5D, Supporting Information). The results indicate that Hsp90 can bind to cytoskeleton proteins associated with EMT. Additionally, Co‐IP also indicated that glycolytic enzymes can interact with cytoskeletal proteins (Figure 5H). This suggests that Hsp90 can form complexes with glycolytic enzymes and cytoskeleton‐associated proteins. We hypothesize that Hsp90 may regulate the distribution of glycolytic metabolic enzymes in tumor cells by binding to cytoskeleton‐related proteins. This would concentrate the enzymes at the cell margin and lamellar pseudopodia, increasing glycolysis levels and providing sufficient energy to promote EMT, migration, and invasion.

To verify the regionalized distribution of glycolytic metabolic enzymes caused by Hsp90 binding to the cytoskeleton, we used immunofluorescence colocalization to visualize the spatial distribution of Hsp90 and metabolic enzymes, and observed the changes in the spatial locations of Hsp90 and metabolic enzymes after Hsp90 inhibitor geldanamycin (GA) treatment. The study provided evidence that Hsp90 inhibition could block the marginal colocalization of Hsp90, ENO1, and PKM2 in tumor cells (Figure 5I and Figure S5E, Supporting Information), moving colocalization closer to the perinuclear region and restoring their uniform distribution in the cytoplasm. And glycolytic levels such as glucose consumption and lactic acid production were also inhibited (Figure 5K and Figure S5G, Supporting Information). At the same time, we investigated whether HSP90 caused different biological energy states and regional distribution, and found that the production and regional distribution of phosphatidylinositol (3,4,5) ‐triphosphate (PIP3) were significantly reduced after inhibition of HSP90, indicating that HSP90 can regulate the production and regional distribution of ATP (Figure S6, Supporting Information). Additionally, blebbistatin treatment inhibited MYH9, resulting in cytoskeleton inhibition, corresponding changes in tumor cell morphology, and disappearance of marginal colocalization of Hsp90, ENO1, and PKM2 in tumor cells (Figure 5J and Figure S4F, Supporting Information). However, blebbistatin treatment did not significantly decrease the level of glycolysis (Figure 5L). It was further demonstrated that Hsp90‐induced regional distribution affects glycolytic metabolic enzymes and depends on cytoskeleton function.

### 2.6 Combination Strategies Targeting Hsp90 and Glycolysis for the Treatment of GC

**Figure 6:**
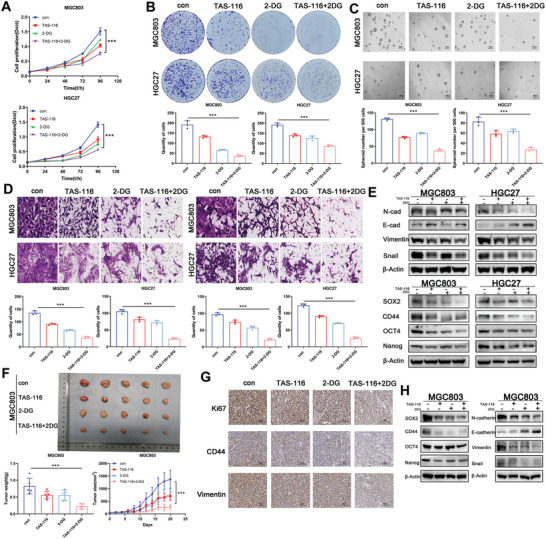
Combination therapy experiment targeting Hsp90 and glycolysis using TAS‐116 and 2‐DG in vivo and in vitro. A) CCK‐8 show cell proliferation capacity of MGC803 and HGC27 after treatment with the Hsp90 inhibitor TAS and the glycolysis inhibitor 2‐DG. B) The colony‐formation ability of MGC803 and HGC27 was detected before and after treatment with the Hsp90 inhibitor TAS‐116 and the glycolysis inhibitor 2‐DG. C) The self‐renewal ability of MGC803 and HGC27 was detected before and after treatment with the Hsp90 inhibitor TAS‐116 and the glycolysis inhibitor 2‐DG. Scale bar, 1000 µm. D) The migration and invasion ability of MGC803 and HGC27 was detected before and after treatment with the Hsp90 inhibitor TAS‐116 and the glycolysis inhibitor 2‐DG. Scale bar, 100 µm. E) Expression of stemness markers and EMT‐related markers was detected before and after treatment with the Hsp90 inhibitor TAS‐116 and the glycolysis inhibitor 2‐DG by western blot. F) In vivo subcutaneous xenograft tumor model of MGC803 with combination therapy in vivo. Tumor appearance, total tumor weights and tumor volumes were assessed. G) Representative IHC staining of Ki‐67, CD44 and Vimentin in xenografted tumors. Scale bar, 100 µm. H) Expression of stemness markers and EMT‐related markers was detected in xenografted tumors. Error bars indicate mean ± SD. *p < 0.05, **p < 0.01 and ***p < 0.001.

Due to the synergistic interaction between Hsp90 and glycolysis, and considering the toxicity associated with Hsp90 inhibitors and glycolysis inhibitors, we propose a combined therapeutic strategy targeting both Hsp90 and glycolysis simultaneously at the molecular level aiming at upstream and downstream components of the involved molecular mechanisms. These include direct targeting of Hsp90 and glycolysis in combination with TAS116 and 2‐DG, and indirect targeting of glycolysis by affecting the cytoskeleton with the MYH9 inhibitor blebbistatin. The results indicated that cell proliferation and self‐renewal ability were significantly inhibited after the combined effect of TAS116 and 2‐DG for 48 hours (**Figure** **6**A–C). The transwell experiment demonstrated a noteworthy decrease in migration and invasion ability (Figure 6D). Additionally, the western blot analysis revealed a significant decrease in the expression levels of the stemness‐related markers after the combination treatment. The changes in the EMT‐related markers indicated an increased potential for EMT (Figure 6E). The cell derived xenograft (CDX) indicated that the combined targeted therapy of TAS116 and 2‐DG had a greater therapeutic effect than any single‐drug treatment. The tumor volume and weight in the mice that treated with the combined drug were significantly lower than those in the mice that treated with a single drug (Figure 6F). The changes in Ki67, vimentin, and CD44 were detected by IHC (Figure 6G). The changes in tumor stemness markers and EMT‐related markers were detected by WB (Figure 6H), further confirming that the combination treatment significantly reduced the proliferation, EMT, and stemness of GC.

**Figure 7:**
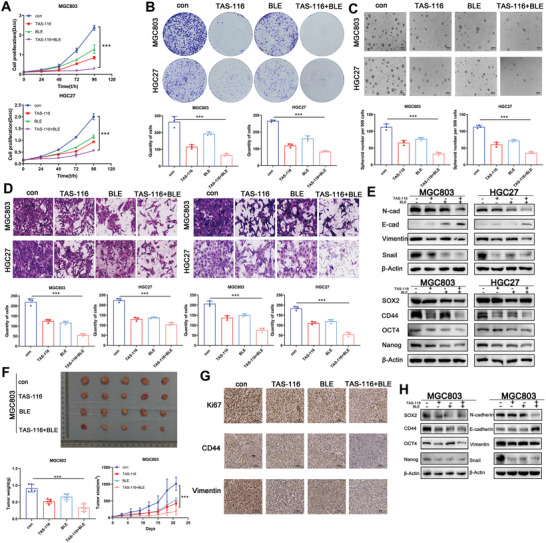
Combination therapy experiment targeting Hsp90 and glycolysis regionalization using TAS‐116 and blebbistatin in vivo and in vitro. A) CCK‐8 show cell proliferation capacity of MGC803 and HGC27 after treatment with the Hsp90 inhibitor TAS‐116 and the MYH9 inhibitor blebbistatin. B) The colony‐formation ability of MGC803 and HGC27 was detected before and after treatment with the Hsp90 inhibitor TAS‐116 and the MYH9 inhibitor blebbistatin. C) The self‐renewal ability of MGC803 and HGC27 was detected before and after treatment with the Hsp90 inhibitor TAS‐116 and the MYH9 inhibitor blebbistatin. Scale bar, 1000 µm. D) The migration and invasion ability of MGC803 and HGC27 was detected before and after treatment with the Hsp90 inhibitor TAS‐116 and the MYH9 inhibitor blebbistatin. Scale bar, 100 µm. E) Expression of stemness markers and EMT‐related markers was detected before and after treatment with the Hsp90 inhibitor TAS‐116 and the MYH9 inhibitor blebbistatin by western blot. F) In vivo subcutaneous xenograft tumor model of MGC803 with combination therapy in vivo. Tumor appearance, total tumor weights and tumor volumes were assessed. G) Representative IHC staining of Ki‐67, CD44 and Vimentin in xenografted tumors. Scale bar, 100 µm. H) Expression of stemness markers and EMT‐related markers was detected in xenografted tumors. Error bars indicate mean ± SD. *p < 0.05, **p < 0.01 and ***p < 0.001.

In addition, when blebbistatin, which indirectly targets glycolysis, was used in combination therapy with TAS‐116, the results also demonstrated that the malignancy‐related biological phenotype and stemness‐related molecular phenotype of GC cells were significantly reduced after combination therapy (**Figure** **7**A–E). In vivo experiments confirmed that tumor volume and weight of mice in the combination treatment group were significantly lower than those in the single drug group (Figure 7F). Proliferation, stemness and EMT‐related markers in tumor tissue were also significantly reduced (Figure 7G,H). It was further confirmed that the combined drug strategy was significantly effective. In conclusion, we suggest that combined targeting of Hsp90 and glycolysis has a positive therapeutic effect in the animal model and may represent a potential clinical strategy for the treatment of GC.

## 3 Discussion

### 3.1 Hsp90 Promotes EMT and a Malignant Phenotype in GC by Regulating Glycolysis Levels

Previous studies have consistently demonstrated Hsp90 overexpression in various tumor tissues, which promotes tumor cell growth.^[^ ^14^, ^18^, ^19^ ^]^ According to our current bioinformatics analysis, Hsp90 was highly expressed in GC tissues, which in combination with glycolysis was able to influence prognosis. Subsequent cellular models further confirmed that Hsp90 promoted EMT and metastasis, thereby regulating the progression and malignant phenotype of GC. Consequently, Hsp90 emerged as a crucial therapeutic target in GC treatment, and inhibitors targeting Hsp90 demonstrated antitumor effects, consistent with previous research findings.

However, most previous studies have primarily focused on the detailed mechanisms by which Hsp90, as a molecular chaperone, promotes tumor progression and regulates the conformation, stability, and function of oncogenic proteins.^[^ ^15b^ ^]^ Delving deeper into our investigation, MS indicated that Hsp90 might interact with glycolytic enzymes. Further validation through coimmunoprecipitation assays and immunofluorescence colocalization confirmed that Hsp90 directly interacted with glycolytic enzymes such as PKM2 and ENO1, exerting direct regulatory control over glycolysis levels. This finding aligns with previous research suggesting that Hsp90 can directly interact with PKM2, increasing the stability of EGFR, and induce Thr‐328 phosphorylation of PKM2, enhancing protein stability, ultimately promoting carcinogenesis.^[^ ^20^ ^]^

The interaction between glycolytic enzymes and Hsp90 appears to be a common phenomenon in different types of tumors, and their interplay may be crucial for tumor progression through various mechanisms, including affecting the expression, stability and activity of glycolytic enzymes.^[^ ^21^, ^22^ ^]^ In this study, by inhibiting glycolysis levels using 2‐DG and examining differentially expressed biological phenotypes of Hsp90, we, for the first time, discovered that the oncogenic function of Hsp90 significantly depends on glycolysis. This implies that Hsp90 can promote the occurrence of tumor EMT and stemness by regulating glycolysis levels. Through the analysis of TCGA clinical samples, we also found that the high expression of Hsp90 aggravated the influence of glycolysis on the prognosis of GC patients, which further verified our conclusion.

### 3.2 Hsp90 Participates in a Multi‐Enzyme Complex to Improve the Efficiency of Glycolysis and Plays a Pro‐Cancer Role

As an abundant cytoplasmic protein, Hsp90 has ATPase activity and interacts with its client proteins in an ATP‐dependent manner to stabilize the epichaperome, the compact and stable protein complex formed by the chaperone. Without the limitation of spatial distance, protein interactions in this complex may be more efficient, thereby helping tumor cells to cope with various stressful environments and promoting tumor cell survival.^[^ ^23^ ^]^

In this study, through Co‐IP and immunofluorescence colocalization, we validated that Hsp90 interacts with a series of glycolytic enzymes involved in the energy production processes of the glycolytic pathway. Unlike previous findings that Hsp90 influences tumor progression by regulating the abundance of PKM,^[^ ^16a^ ^]^ we found that the differential expression of Hsp90 did not affect the expression and activity of metabolic enzymes in MGC803 and HGC27. Moreover, Hsp90 forms complexes with glycolytic enzymes through its unique chaperone structure. The formation of this enzyme complex relies on the presence of Hsp90, facilitating the rapid progression of glycolytic intermediates to the enzymes involved in the downstream biochemical reactions of the glycolytic cascade. This accelerates the transfer of intermediate products along the glycolytic cascade, thereby enhancing the overall efficiency of glycolysis. Consequently, Hsp90 promotes carcinogenesis by elevating glycolytic levels, even in instances where the content and activity of metabolic enzymes remain unaltered.

Interestingly, it has been reported that the oncogenic form of Hsp90 can form advanced complexes with the HSP70 chaperone group, termed chaperonin containing T‐complex (CCT), which is more stable in its active conformation than classical chaperonin complexes. By restricting the cytoplasmic distribution of functionally relevant enzymes, this assembly forms functional microcompartments or “metabolons” enhancing the efficiency of metabolic pathways and providing a metabolic advantage for maintaining biomass and nutrient utilization.^[^ ^24^ ^]^ This suggests the potential of Hsp90 to assemble multiple enzyme complexes in the context of glycolytic metabolism. Additionally, the permissive assembly of the PGK1‐PGAM1‐ENO1‐PKM2‐LDHA complex through gLINC has been shown to enhance glycolytic capacity.^[^ ^25^ ^]^ Such complexes aid in “channeling” metabolites between consecutive enzymes, promoting catalytic efficiency and facilitating cancer cell growth.^[^ ^26^ ^]^ This aligns with our proposition that Hsp90 forms multi‐enzyme complexes to promote glycolytic efficiency from a biochemical reaction perspective, thereby increasing intracellular glycolytic levels and generating abundant ATP and lactate.

Moreover, recent research indicates that elevated glycolytic levels can enhance drug resistance in various cancers, such as lung cancer, breast cancer, and colon cancer.^[^ ^27^ ^]^ The accumulation of glycolytic products such as ATP^[^ ^28^ ^]^ and lactate in the cytoplasm^[^ ^29^ ^]^ can influence signaling pathways such as AMPK/mTOR and PI3K/AKT/mTOR, thereby promoting tumor development and regulating cancer stem cell‐associated characteristics.^[^ ^30^ ^]^ This provides theoretical support for the notion that Hsp90's formation of multi‐enzyme complexes to enhance glycolytic efficiency contributes to its oncogenic function.

### 3.3 Hsp90 Can Combine with Cytoskeletal Protein to Regulate the Regional Distribution of Glycolytic Enzyme Complex, Thereby Improving Glycolysis at the Cell Edge and Further Promoting the Metastasis of Tumor Cells

Additionally, we propose for the first time that glycolysis can exert its oncogenic function by altering its intracellular localization. Our findings revealed that the proteins interacting with Hsp90 include cytoskeleton proteins such as actin and myosin, which are associated with EMT.^[^ ^31^ ^]^ Actin‐rich membrane processes promote cell movement and act as extensions of the cytoskeleton. Actin‐rich pseudopodia perform proteolytic functions in extracellular matrix (ECM) degradation, thereby promoting cell invasion and EMT.^[^ ^32^ ^]^ As a microfilament cytoskeletal protein, MYH9 is involved in cell adhesion, migration, and invasion, regulation of cell signaling and transport, and promotion of EMT, and can use spatial dynamics to regulate cell states.^[^ ^33^ ^]^ Through immunofluorescence observations, we noted that Hsp90, multienzyme complexes, and cytoskeleton proteins were primarily distributed at the cell margin and pseudopodia. Previous studies have started unveiling potential interactions between cellular metabolic activities and the cytoskeleton.^[^ ^11^, ^34^ ^]^ Many glycolytic enzymes are associated with the cytoskeleton and can be regulated by actin dynamics. Inhibition of PI3K has been shown to limit glycolytic flux by binding aldolase to the actin cytoskeleton. When the actin cytoskeleton is disrupted, filamentous actin binds to aldolase, releasing it and increasing aldolase activity.^[^ ^11^ ^]^ This implies that cytoskeleton dynamics may influence glycolytic enzyme storage and release, thereby affecting glycolysis levels.

Recent research has also demonstrated mechanisms by which glycolysis responds to the structural features of the actin cytoskeleton. This coupling of cellular metabolism with the mechanical properties of surrounding tissues reveals that the structural regulation of the actin cytoskeleton controls glycolytic reactions.^[^ ^35^ ^]^ Building on this, we propose that glycolytic enzymes not only rely on Hsp90 for assembling multi‐enzyme complexes but also bind to cytoskeleton proteins via Hsp90 as a hub. This interaction can modify the regional distribution of glycolytic enzyme complexes within tumor cells, concentrating glycolytic enzymes at the edges and in pseudopodia of tumor cells, thereby facilitating efficient energy utilization. Immunofluorescence further confirmed that the Hsp90 inhibitor GA and the cytoskeleton inhibitor blebbistatin respectively changed the formation and regional distribution characteristics of glycolytic enzyme complexes. In addition, according to previous studies, we detected PIP3, which indirectly indicates the production and regionalization of ATP energy in the cell by immunofluorescence,^[^ ^28^, ^36^ ^]^ providing more evidence supporting the hypothesis that Hsp90 contributes to the regionalization of glycolytic distribution, thereby enabling localized energy supply within key subcellular regions of tumor cells.

This proposition also indirectly indicates that the oncogenic effect of glycolysis is partially dependent on the formation of glycolytic enzyme complexes and their regional distribution in crucial cytoplasmic locations. This allows glycolysis to occur more concentratedly in energetically demanding key regions such as pseudopodia and the edges of tumor cells, thereby generating sufficient intracellular ATP to enhance ATP‐driven biochemical/enzymatic reactions. This, in turn, activates receptor tyrosine kinases and phosphorylates proteins in PI3K‐related pathways on the cell membrane.^[^ ^28^, ^36^, ^37^ ^]^ Subsequently, this drives the release of proteases (such as MMPs and cathepsins) and regulates the formation of actin‐rich structures, such as filopodia, thereby further promoting EMT and metastasis.^[^ ^38^ ^]^ This provides a new explanation for how glycolysis promotes the occurrence of EMT and metastasis in tumors.

### 3.4 Summary and Research Limitations

In this study, we investigated the regulatory effect and specific mechanism of Hsp90 on the stemness of GC cells. By combining with glycolytic enzymes such as ENO1 and PKM2 to form multi‐enzyme complexes, Hsp90 improved glycolytic efficiency, thereby promoting tumor proliferation and stemness. Additionally, in some highly invasive tumor cells with high EMT status, Hsp90 could also combine with cytoskeleton‐related proteins such as MYH9 and actin to alter the cytoplasmic distribution of glycolytic metabolic enzyme complexes in tumor cells. It concentrated at the cell edge and pseudopodia, thereby improving the level of glycolysis at the cell edge, generating a large amount of energy supply to promote tumor cells to develop EMT and further promoting tumor migration, invasion, and stemness, and form a malignant heterogeneous phenotype with positive feedback.

Due to current limitations in technological methodologies, the precise composition of the multi‐enzyme complex has not yet been definitively determined, and we have preliminarily verified the existence of key enzymes in the production stage of glycolysis, thereby clarifying our conclusions. However, we are unable to substantiate and quantify the enhancement in enzymatic reaction efficiency with specific data. Present evidence primarily relied on immunofluorescence colocalization to illustrate the regional distribution of glycolytic enzymes and employed inhibitors to demonstrate their impact on the malignant phenotype of tumors. In our subsequent investigations, we plan to employ subcellular structural‐level detection methods, such as probe labeling, to comprehensively validate the formation of multi‐enzyme complexes and the regionalization of glycolysis. This approach aims to further elucidate the mechanistic impact of multi‐enzyme complexes on tumor progression.

HSP90 inhibitors are widely used in various types of oncology clinical trials due to the important role of HSP90 in tumor regulation.^[^ ^39^ ^]^ However, many Hsp90 inhibitors that have entered clinical trials have been deemed clinical failures due to low efficacy, toxicity, or drug resistance.^[^ ^40^ ^]^ Nevertheless, the proportion of drug combinations involving Hsp90 inhibitors with other antineoplastic drugs or oncoprotein inhibitors has significantly increased.^[^ ^41^ ^]^ Clinical trials have demonstrated that combining Hsp90 inhibitors with other inhibitors produces synergistic effects and anti‐tolerance properties.^[^ ^42^ ^]^ The field of oncology has shown interest in the development of dual inhibitors of Hsp90 and the search for a combination drug strategy.^[^ ^43^ ^]^ In this study, we identified the possibility of combining the Hsp90 inhibitor TAS116 and the glycolysis inhibitor 2‐DG for targeted therapy, and combining the Hsp90 inhibitor TAS116 and the MYH9 inhibitor blebbistatin for targeted therapy of GC. Nevertheless, given that cell and nude mouse models may not fully recapitulate the complexity of the human tumor microenvironment, our results require further validation. Our study also offers potential combination drug strategies and new therapeutic ideas for clinical practice.

## 4 Conclusions

We demonstrated that Hsp90 can be used as a tumor therapeutic target to regulate the stem‐like properties and malignant phenotypes of cancer cells. The effect of Hsp90 on the reprogramming of glucose metabolism in tumor cells and its molecular mechanism were further investigated. We have demonstrated, for the first time, the pivotal role of Hsp90 in orchestrating glycolysis and promoting tumor cell stemness and EMT. Our findings elucidate how Hsp90 regulates glycolysis by interacting with glycolytic enzymes, particularly PKM2 and ENO1, to form multi‐enzyme complexes. Additionally, Hsp90 influences the distribution of crucial cytoplasmic regions through its interaction with cytoskeleton‐related proteins. This orchestrated regulation enhances glycolytic efficiency, facilitating optimal energy utilization in cytoplasmic regions with heightened energy demands. This study provides novel targets and therapeutic ideas for the treatment of GC.

## 5 Experimental Section

### Cell Lines and Culture

Established human GC cell lines MGC803 and HGC27 were obtained from the Chinese Academy of Sciences (China) and cultured in DMEM with 10% FBS. The cell lines were maintained in a humidified atmosphere with 5% CO_2_ at 37 °C. Gastric cancer stem cells (GCSCs) were respectively enriched from MGC803 and HGC27 cells, as described previously.^[^ ^44^ ^]^

### Bioinformatics Analysis

Gene expression data were extracted from The Cancer Genome Atlas (TCGA) database. According to p‐value < 0.05, | fold change | > 1.5, the “limma” package was used to perform the differentially expressed genes (DEGs) selection. The gene set associated with glycolysis pathway (HALLMARK_GLYCOLYSIS) was searched in the Molecular Signature Database (MSigDB) v4.0. Gene set enrichment analysis (GSEA) was employed to characterize the DEGs related to glycolysis, aiming to identify the signaling pathways and networks potentially involved in the progression of GC. The study screened a total of 109 glycolytic‐related genes (GRGs) from 7623 mRNA expression profiles. Venn diagrams were employed. The “glmnet” package is used to perform the least absolute shrinkage and selection operator (LASSO) regression, and the optimal value of the penalty parameter *λ* is determined by cross‐validation. Multivariate Cox regression analysis was performed using the “survival” package. The coefficient (Coefi) and expressions (Expri) of prospective prognostic GRGs were used to estimate GRG scores for each stomach adenocarcinoma (STAD) patients, and the scoring formula was $\sum_{i\;=\;1}^{n}\mathrm{Coefi}\;\times \;\mathrm{Expri}$. Kaplan–Meier (KM) curves were plotted to compare the overall survival (OS) of each group.

### Cell Transfection for Silencing and Overexpression of Hsp90

To silence Hsp90, the two cell lines were infected with lentivirus containing shRNA targeting Hsp90 (#1 GTTATCCTACACCTGAAAGAA, #2 TACTTGGAGGAACGAAGAATA). After 2 d, the cell lines stably expressing Hsp90 knockdown (shHsp90) were enriched by 2 d puromycin (Sigma, USA) treatment for selecting positive clones. To achieve transient overexpression of Hsp90 (oeHsp90), cells were transfected with the pcSLenti‐CMV‐Hsp90AA1‐3xFLAG‐PGK‐Puro‐WPRE3 plasmid. Additionally, a blank plasmid was transfected as a negative control. The preparation and the titer detection of the overexpressed plasmid and the shRNA plasmid‐encoded lentivirus were provided by Obio Technology (Shanghai).

### Cell Invasion and Migration Assay

To evaluate the migratory and invasive activity, a Transwell chamber (24‐well insert; pore size, 8 µm; Corning, USA) was uncoated or coated with diluted Matrigel (BD Biosciences, USA). 2×104 serum‐starved cells were inoculated into the upper chamber in serum‐free medium. After incubation at 37 °C for 24 h, the invaded cells were fixed, stained, and counted. The quantitative analysis of cell migration and invasion was performed.

### Colony‐Formation Assay and Sphere‐Formation Assay

The cells were seeded in a six‐well plate (500 cells/well) for each experimental group and cultured in 5% CO_2_ at 37 °C for 2 weeks. The clones were then stained with 0.1% crystal violet (Solarbio, China) for 30 min following the fixation in 4% paraformaldehyde (Solarbio, China). Colony numbers were counted and photographed by a camera. The self‐renewal capacity was evaluated by a sphere‐formation assay, which was performed as described previously.^[^ ^9^ ^]^

### Western Blot (Wb)

Western blot analysis was performed following standard procedure as previously described.^[^ ^21^ ^]^ The primary antibodies to Hsp90 (ab13492), β‐actin (4970S), CD44 (ab157107), SOX2 (ab97959), Oct4 (ab18976), and ENO1 (ab227978) were from Abcam (USA), and those to vimentin (5741S), N‐cadherin (13116S), E‐cadherin (3195S), Snail (3879S), and Nanog (3580S) were from Cell Signaling Technology, Inc. (USA). The additional antibodies GAPDH (Cat No: 10494‐1‐AP) and PKM2 (Cat No. 15822‐1‐AP) were from Proteintech (China). Secondary antibodies were HRP‐labeled goat anti‐rabbit and anti‐mouse IgG (Jackson, USA).

### Flow Cytometry for Detection

The cells in different treatment groups were incubated with CD44 (eBioscience, 15‐0441‐82, USA), CD90 (eBioscience, 12‐0909‐42, USA), and homologous control at 37 °C for 1 h. The cells were washed with PBS three times after each reaction to detect the ratio of CD44 and CD90 expression by Attune NxT Flow Cytometer (Thermo Fisher, USA).

### Cell Proliferation Assay

The cells were seeded in 96‐well plates (4000 cells per well) and incubated overnight. Then, the cells were treated with TAS‐116 (MCE, HY‐15785, USA), 2‐deoxy‐D‐glucose (2‐DG, MCE, HY‐13966, USA), blebbistatin (Selleck, S7099, USA), and PBS for cell proliferation analysis. Absorbance at 450 nm was determined by a microplate reader every 24 h, for a total of 96 h.

### Glucose Consumption and Lactic Acid Measurement

The cell culture supernatant was collected after drug treatment. Glucose consumption by the cells was measured by colorimetry following the instructions of a Glucose and Sucrose Assay Kit (Sigma‐Aldrich, MAK013, USA). Subsequently, lactic acid production was measured using a Lactate Colorimetric Assay Kit II (Abcam, ab65331, UK). The cells were then collected and counted. Glucose consumption and lactate production were standardized by the number of cells (µmol/106 cells).

### Adenosine 5′‐Triphosphate (ATP) Production Measurement

The cells were collected after drug treatment, and ATP production by the cells was measured by a chemiluminescence assay with ATP detection kit (Beyotime, S0027, China). The remaining cells were collected for protein extraction and tested for protein content. The ATP production was normalized to the control (nmol per mg protein).

### Extracellular Acidification Rate (ECAR) Measurement

Cells were inoculated into XF96 plates at 15 000 cells per well and cultured overnight. The cells were then washed and placed in a CO2‐free incubator at 37 °C for 60 min Glucose (10 × 10^−3^ m), oligomycin (1 × 10^−6^ m), and 2‐deoxyglucose (2‐DG, 50 × 10^−3^ m) were then added in sequence to detect ECAR at a given time point. Finally, ECAR was tested to evaluate glycolytic fluxes using a Seahorse XFe96 Analyzer (Agilent Technologies Inc., USA).

### RNA Isolation and Quantitative Real‐Time PCR (q‐PCR)

The expression of Hsp90, stemness‐related markers and glycolytic enzymes in GC cells was confirmed by qRT‐PCR. The reagents and methods used in the details of RNA extraction and PCR experiments are described previously.^[^ ^45^ ^]^ Finally, the mRNA expression of the target genes was quantified using a comparative threshold cycle method (2^−ΔΔCT^). The PCR primer sequences are listed in Table S1 (Supporting Information).

### Co‐Immunoprecipitation (Co‐IP) and Mass Spectrometry Assays

Immunoprecipitation (IP) buffer containing 50 × 10^−3^ m Tris pH 7.5, 150 × 10^−3^ m NaCl and 1% NP‐40 was used to prepare cell lysates from 5 × 106 cells. Hsp90 (Proteintech, Cat No. 13171‐1‐AP) antibody (2 µg) was added to Protein A/G Magnetic Beads (MCE, Cat. No. HY‐K0202) in binding/wash buffer with IgG (Proteintech, Cat No. 30000‐0‐AP) antibody as the negative control group. The reaction was incubated for 30 min at room temperature on a rotator. The protein lysate was added mixed with magnetic bead–antibody complex and incubated O/N at 4 °C. Proteins were denatured by boiling at 95 °C for 5 min using 1× SDS‐PAGE Protein Loading Buffer (Solarbio, #P1040). IP samples were used for SDS‐PAGE and WB analyses. The proteomics studies were carried out at the mass spectrometry (MS) Core Facility at the Institute of Microbiology, Chinese Academy of Sciences (IMCAS).

### Immunofluorescence and Immunohistochemistry (IHC)

Cells were inoculated into the 24‐well plate at a density of 5×104. The cells were fixed, permeabilized, and blocked after cell attachment. Subsequent incubation with primary and secondary antibodies (Dylight 488, AlexaFluor 647) against Hsp90, ENO1 and PKM2. Nucleus staining with DAPI. Images were captured by confocal laser scanning microscopy (Leica Microsystems, Wetzlar, Germany).

As mentioned above, IHC staining was performed on tumor tissues of nude mice.^[^ ^9^ ^]^ The following antibodies were used: Hsp90 (Cat No. 13171‐AP; Proteintech), Ki67 (Cat No. 27309‐1‐AP; Proteintech), CD44 (#ab157107; Abcam), and Vimentin (#5741S, CST). For quantitative analysis, each IHC‐stained slice was photographed at five different random regions, and Aperio Image‐Scope 12.4 was used to analyze the expression levels of Hsp90, Ki67, CD44, and vimentin.

### Tumorigenicity in BALB/C Nude Mice

All ethics committee‐approved protocols were followed for all animal studies (Ethics). BALB/c nude mice (4–5 weeks old) were obtained from HFK Biosciences (China). 5×106 cells were injected subcutaneously into the left axilla or back of nude mice to induce tumors (5 mice per group). The mice were administered with TAS‐116 (MCE, HY‐15785, USA), 2‐deoxy‐D‐glucose (MCE, HY‐13966, USA), blebbistatin (Selleck, S7099, USA), and vehicle control. The size of the tumors was measured every 3 d. After 21 d, the mice were euthanized and the tumor xenografts were harvested, photographed, and weighed in each group. Tumor xenograft tissues were also used to perform IHC and western blot analysis for stemness markers.

### Statistical Analysis

All data are shown as mean ± standard deviations (SD) or number (percentage, %) of at least three independent experiments. Chi‐squared test or Student's t‐test was used to compare the two groups. One‐way analysis of variance was used to compare multiple groups. Survival was analyzed by the Kaplan‐Meier method and compared by the log‐rank test. R version 4.2.0, SPSS 25.0 and GraphPad Prism 8.0 were used for data analysis and visualization. Data were considered statistically significant when the *P* < 0.05 (*, *P* < 0.05; **, *P* < 0.01; and ***, *P* < 0.001).

### Ethics Approval Statement

All animal experiments were approved by animal care and use committee of Beijing Jishuitan Hospital, Beijing, China (Ethical approval number: 2023‐04‐04).

## Conflict of Interest

The authors declare no conflict of interest.

## Supporting information

Supporting Information

## Data Availability

The data that support the findings of this study are available from the corresponding author upon reasonable request.

## References

[1] a) H. Sung , J. Ferlay , R. L. Siegel , M. Laversanne , I. Soerjomataram , A. Jemal , F. Bray , Ca‐Cancer J. Clin. 2021, 71, 209; 33538338 10.3322/caac.21660

[2] A. P. Thrift , H. B. El‐Serag , Clin. Gastroenterol. Hepatol. 2020, 18, 534. 31362118 10.1016/j.cgh.2019.07.045 PMC8859863

[3] M. Tokunaga , Y. Sato , M. Nakagawa , T. Aburatani , T. Matsuyama , Y. Nakajima , Y. Kinugasa , Surg. Today 2020, 50, 30. 31612329 10.1007/s00595-019-01896-5 PMC6954129

[4] a) H. H. Hartgrink , E. P. Jansen , N. C. van Grieken , C. J. van de Velde , Lancet 2009, 374, 477; 19625077 10.1016/S0140-6736(09)60617-6 PMC4613761

[5] a) S. S. Joshi , B. D. Badgwell , Ca‐Cancer J. Clin. 2021, 71, 264; 33592120 10.3322/caac.21657 PMC9927927

[6] a) S. Y. Lunt , M. G. Vander Heiden , Annu. Rev. Cell Dev. Biol. 2011, 27, 441; 21985671 10.1146/annurev-cellbio-092910-154237

[7] a) B. Bhattacharya , M. F. Mohd Omar , R. Soong , Br. J. Pharmacol. 2016, 173, 970; 26750865 10.1111/bph.13422 PMC4793921

[8] a) A. M. Di Francesco , A. Toesca , C. Cenciarelli , A. Giordano , A. Gasbarrini , M. A. Puglisi , J. Cell. Physiol. 2016, 231, 2081; 26791139 10.1002/jcp.25318

[9] T. Yang , X. Shu , H. W. Zhang , L. X. Sun , L. Yu , J. Liu , L. C. Sun , Z. H. Yang , Y. L. Ran , Cell Death Dis. 2020, 11, 870. 33067426 10.1038/s41419-020-03087-4 PMC7567818

[10] a) J. Lin , W. Fang , Z. Xiang , Q. Wang , H. Cheng , S. Chen , J. Fang , J. Liu , Q. Wang , Z. Lu , L. Ma , Front. Immunol. 2023, 14, 1189953; 37377974 10.3389/fimmu.2023.1189953 PMC10291184

[11] H. Hu , A. Juvekar , C. A. Lyssiotis , E. C. Lien , J. G. Albeck , D. Oh , G. Varma , Y. P. Hung , S. Ullas , J. Lauring , P. Seth , M. R. Lundquist , D. R. Tolan , A. K. Grant , D. J. Needleman , J. M. Asara , L. C. Cantley , G. M. Wulf , Cell 2016, 164, 433. 26824656 10.1016/j.cell.2015.12.042 PMC4898774

[12] S. Papadaki , A. Magklara , Cancers 2022, 14, 5912. 36497394 10.3390/cancers14235912 PMC9741285

[13] a) J. Sanchez , T. R. Carter , M. S. Cohen , B. S. J. Blagg , Curr. Cancer Drug Targets 2020, 20, 253; 31793427 10.2174/1568009619666191202101330 PMC7502213

[14] a) F. H. Schopf , M. M. Biebl , J. Buchner , Nat. Rev. Mol. Cell Biol. 2017, 18, 345; 28429788 10.1038/nrm.2017.20

[15] a) X. Liu , S. Chen , J. Tu , W. Cai , Q. Xu , Int. J. Mol. Med. 2016, 37, 825; 26846697 10.3892/ijmm.2016.2482

[16] a) Q. Xu , J. Tu , C. Dou , J. Zhang , L. Yang , X. Liu , K. Lei , Z. Liu , Y. Wang , L. Li , H. Bao , J. Wang , K. Tu , Mol. Cancer 2017, 16, 178; 29262861 10.1186/s12943-017-0748-y PMC5738801

[17] a) B. Pajak , E. Siwiak , M. Soltyka , A. Priebe , R. Zielinski , I. Fokt , M. Ziemniak , A. Jaskiewicz , R. Borowski , T. Domoradzki , W. Priebe , Int. J. Mol. Sci. 2019, 21, 234; 31905745 10.3390/ijms21010234 PMC6982256

[18] a) L. Li , L. Wang , Q. D. You , X. L. Xu , J. Med. Chem. 2020, 63, 1798; 31663736 10.1021/acs.jmedchem.9b00940

[19] S. Maiti , D. Picard , Biomolecules 2022, 12, 1166. 36139005 10.3390/biom12091166 PMC9496497

[20] Y. C. Yang , T. Y. Cheng , S. M. Huang , C. Y. Su , P. W. Yang , J. M. Lee , C. K. Chen , M. Hsiao , K. T. Hua , M. L. Kuo , Oncogene 2016, 35, 3387. 26500058 10.1038/onc.2015.397

[21] X. Shu , K. Y. Cao , H. Q. Liu , L. Yu , L. X. Sun , Z. H. Yang , C. A. Wu , Y. L. Ran , Stem Cell Res. Ther. 2021, 12, 119. 33579362 10.1186/s13287-021-02160-9 PMC7881626

[22] a) X. Xu , Y. Chen , S. Shao , J. Wang , J. Shan , Y. Wang , Y. Wang , J. Chang , T. Zhou , R. Chen , S. Liu , C. Li , C. Li , X. Li , Int. J. Biol. Sci. 2024, 20, 1492; 38385089 10.7150/ijbs.90774 PMC10878141

[23] N. Pillarsetty , K. Jhaveri , T. Taldone , E. Caldas‐Lopes , B. Punzalan , S. Joshi , A. Bolaender , M. M. Uddin , A. Rodina , P. Yan , A. Ku , T. Ku , S. K. Shah , S. Lyashchenko , E. Burnazi , T. Wang , N. Lecomte , Y. Janjigian , A. Younes , C. W. Batlevi , M. L. Guzman , G. J. Roboz , J. Koziorowski , P. Zanzonico , M. L. Alpaugh , A. Corben , S. Modi , L. Norton , S. M. Larson , J. S. Lewis , et al., Cancer Cell 2019, 36, 559. 31668946 10.1016/j.ccell.2019.09.007 PMC6996250

[24] a) A. Rodina , T. Wang , P. Yan , E. D. Gomes , M. P. Dunphy , N. Pillarsetty , J. Koren , J. F. Gerecitano , T. Taldone , H. Zong , E. Caldas‐Lopes , M. Alpaugh , A. Corben , M. Riolo , B. Beattie , C. Pressl , R. I. Peter , C. Xu , R. Trondl , H. J. Patel , F. Shimizu , A. Bolaender , C. Yang , P. Panchal , M. F. Farooq , S. Kishinevsky , S. Modi , O. Lin , F. Chu , S. Patil , et al., Nature 2016, 538, 397; 27706135 10.1038/nature19807 PMC5283383

[25] Y. Zhu , L. Jin , R. Shi , J. Li , Y. Wang , L. Zhang , C. Z. Liang , V. K. Narayana , D. P. De Souza , R. F. Thorne , L. R. Zhang , X. D. Zhang , M. Wu , Mol. Cell 2022, 82, 542. 35081364 10.1016/j.molcel.2021.11.017

[26] F. Wu , S. Minteer , Angew. Chem., Int. Ed. Engl. 2015, 54, 1851. 25537779 10.1002/anie.201409336

[27] a) M. Fanciulli , T. Bruno , A. Giovannelli , F. P. Gentile , M. Di Padova , O. Rubiu , A. Floridi , Clin. Cancer Res. 2000, 6, 1590; 10778993

[28] a) K. Xu , N. Yin , M. Peng , E. G. Stamatiades , A. Shyu , P. Li , X. Zhang , M. H. Do , Z. Wang , K. J. Capistrano , C. Chou , A. G. Levine , A. Y. Rudensky , M. O. Li , Science 2021, 371, 405; 33479154 10.1126/science.abb2683 PMC8380312

[29] a) F. Hirschhaeuser , U. G. Sattler , W. Mueller‐Klieser , Cancer Res. 2011, 71, 6921; 22084445 10.1158/0008-5472.CAN-11-1457

[30] F. Gao , Y. Tang , W. L. Liu , M. Z. Zou , C. Huang , C. J. Liu , X. Z. Zhang , Adv. Mater. 2019, 31, 1904639. 10.1002/adma.201904639 31692128

[31] a) S. Lamouille , J. Xu , R. Derynck , Nat. Rev. Mol. Cell Biol. 2014, 15, 178; 24556840 10.1038/nrm3758 PMC4240281

[32] M. A. McNiven , Trends Cell Biol. 2013, 23, 16. 22999190 10.1016/j.tcb.2012.08.009 PMC3905740

[33] a) B. Yang , H. Liu , Y. Bi , C. Cheng , G. Li , P. Kong , L. Zhang , R. Shi , Y. Zhang , R. Zhang , X. Cheng , Int. J. Med. Sci. 2020, 17, 2013; 32788880 10.7150/ijms.46234 PMC7415390

[34] a) W. J. Sullivan , P. J. Mullen , E. W. Schmid , A. Flores , M. Momcilovic , M. S. Sharpley , D. Jelinek , A. E. Whiteley , M. B. Maxwell , B. R. Wilde , U. Banerjee , H. A. Coller , D. B. Shackelford , D. Braas , D. E. Ayer , T. Q. de Aguiar Vallim , W. E. Lowry , H. R. Christofk , Cell 2018, 175, 117; 30197082 10.1016/j.cell.2018.08.017 PMC6151140

[35] J. S. Park , C. J. Burckhardt , R. Lazcano , L. M. Solis , T. Isogai , L. Li , C. S. Chen , B. Gao , J. D. Minna , R. Bachoo , R. J. DeBerardinis , G. Danuser , Nature 2020, 578, 621. 32051585 10.1038/s41586-020-1998-1 PMC7210009

[36] K. Xu , N. Yin , M. Peng , E. G. Stamatiades , S. Chhangawala , A. Shyu , P. Li , X. Zhang , M. H. Do , K. J. Capistrano , C. Chou , C. S. Leslie , M. O. Li , Immunity 2021, 54, 976. 33979589 10.1016/j.immuni.2021.04.008 PMC8130647

[37] M. Zhang , H. Jang , R. Nussinov , Chem. Sci. 2019, 10, 3671. 30996962 10.1039/c8sc04498h PMC6430085

[38] a) Q. Zhang , P. Feng , X. H. Zhu , S. Q. Zhou , M. L. Ye , X. J. Yang , S. Gong , S. Y. Huang , X. R. Tan , S. W. He , Y. Q. Li , Cell Death Dis. 2023, 14, 697; 37875476 10.1038/s41419-023-06225-w PMC10598267

[39] a) A. Mielczarek‐Lewandowska , M. L. Hartman , M. Czyz , Apoptosis 2020, 25, 12; 31659567 10.1007/s10495-019-01577-1 PMC6965345

[40] a) L. Li , N. N. Chen , Q. D. You , X. L. Xu , Expert Opin. Ther. Pat. 2021, 31, 67; 32990109 10.1080/13543776.2021.1829595

[41] a) K. Kryeziu , J. Bruun , T. K. Guren , A. Sveen , R. A. Lothe , Biochim. Biophys. Acta, Rev. Cancer 2019, 1871, 240; 30708039 10.1016/j.bbcan.2019.01.002

[42] X. Lu , L. Xiao , L. Wang , D. M. Ruden , Biochem. Pharmacol. 2012, 83, 995. 22120678 10.1016/j.bcp.2011.11.011 PMC3299878

[43] X. Xie , N. Zhang , X. Li , H. Huang , C. Peng , W. Huang , L. J. Foster , G. He , B. Han , Bioorg. Chem. 2023, 139, 106721. 37467620 10.1016/j.bioorg.2023.106721

[44] S. Takaishi , T. Okumura , S. Tu , S. S. Wang , W. Shibata , R. Vigneshwaran , S. A. Gordon , Y. Shimada , T. C. Wang , Stem Cells 2009, 27, 1006. 19415765 10.1002/stem.30 PMC2746367

[45] P. Zhan , X. Shu , M. Chen , L. Sun , L. Yu , J. Liu , L. Sun , Z. Yang , Y. Ran , Life Sci. 2021, 276, 119405. 33798550 10.1016/j.lfs.2021.119405

