# Supplementary material for: Hsp90 Promotes Gastric Cancer Cell Metastasis and Stemness by Regulating the Regional Distribution of Glycolysis‐Related Metabolic Enzymes in the Cytoplasm
Source: Adv Sci (Weinh). 2024 Jun 14;11(33):2310109. doi: 10.1002/advs.202310109 (PMC11434123; doi:10.1002/advs.202310109)
Supplement: Supplementary file 1 — Supporting Information [file ADVS-11-2310109-s001.docx]

**Supporting Information**

**Title：**Hsp90 promotes gastric cancer cell metastasis and stemness by regulating the regional distribution of glycolysis-related metabolic enzymes in the cytoplasm

Shiya Liu, Gaigai Shen, Xuanyu Zhou, Lixin Sun, Yu Long, Yuanting Cao, Xiong Shu*, Yuliang Ran*

**Content**

Supplementary Figures (Figure S1-S6)

Supplementary Table (Table S1)

**
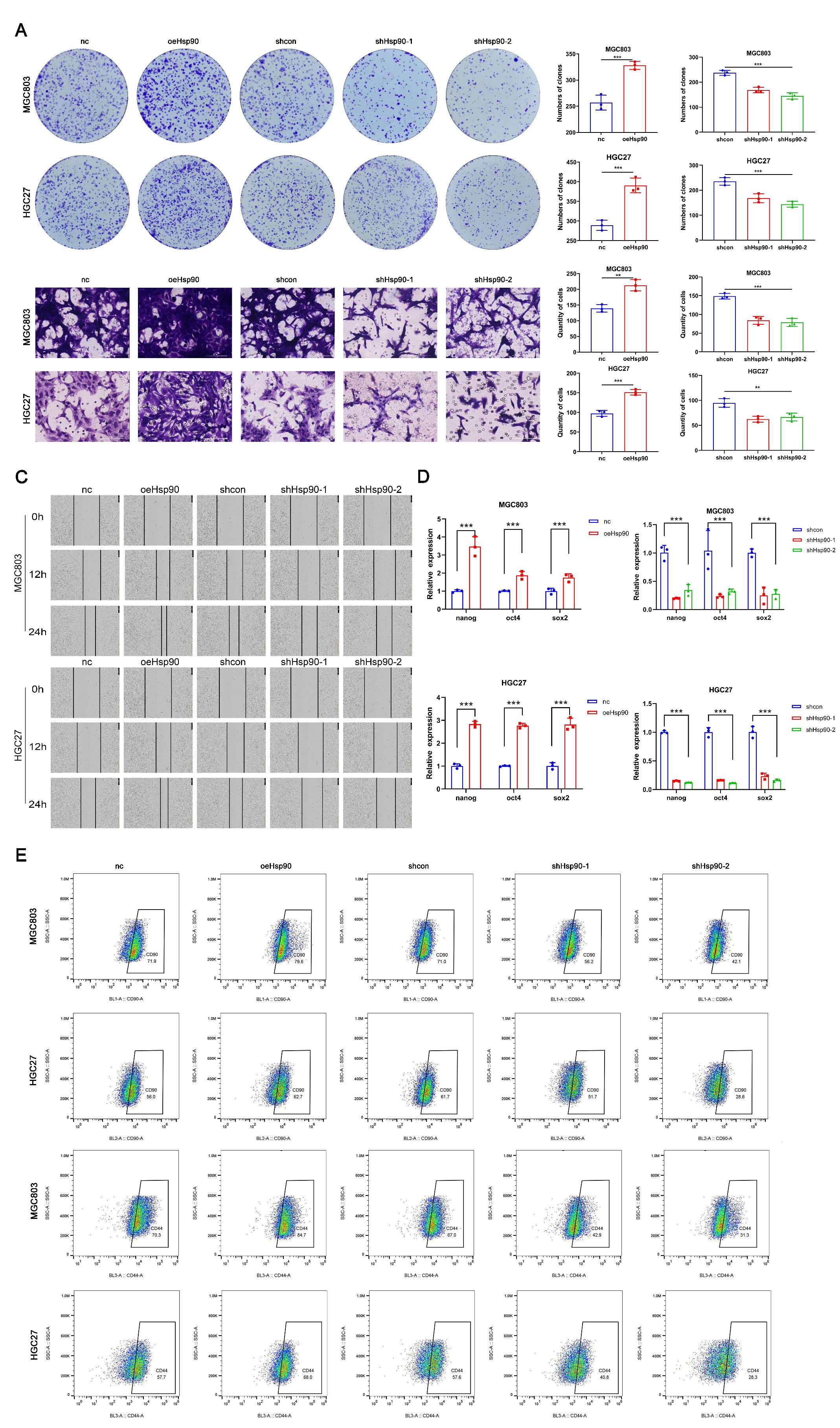
**

**Figure S1.** High Hsp90 promotes proliferation, metastasis and other stem-related features of gastric cancer.

1. Analysis of the colony-formation abilities of MGC803 and HGC27 cells stably expressing nc, oeHsp90, shcon, or shHsp90.
2. Analysis of the migration abilities of MGC803 and HGC27 cells stably expressing nc, oeHsp90, shcon, or shHsp90. Scale bar, 100 μm.
3. The migration abilities of MGC803 and HGC27 stable cell lines was detected by plate scratch test. Scale bar, 100 μm.
4. Expression of stemness markers in MGC803 and HGC27 stable cell lines was detected by Quantitative RT‐PCR.
5. The proportion of CD44+ and CD90+ tumor stem cells in MGC803 and HGC27 stable cell lines was detected by flow cytometry.

Error bars indicate mean ± SD. *p < 0.05, **p < 0.01 and ***p < 0.001.


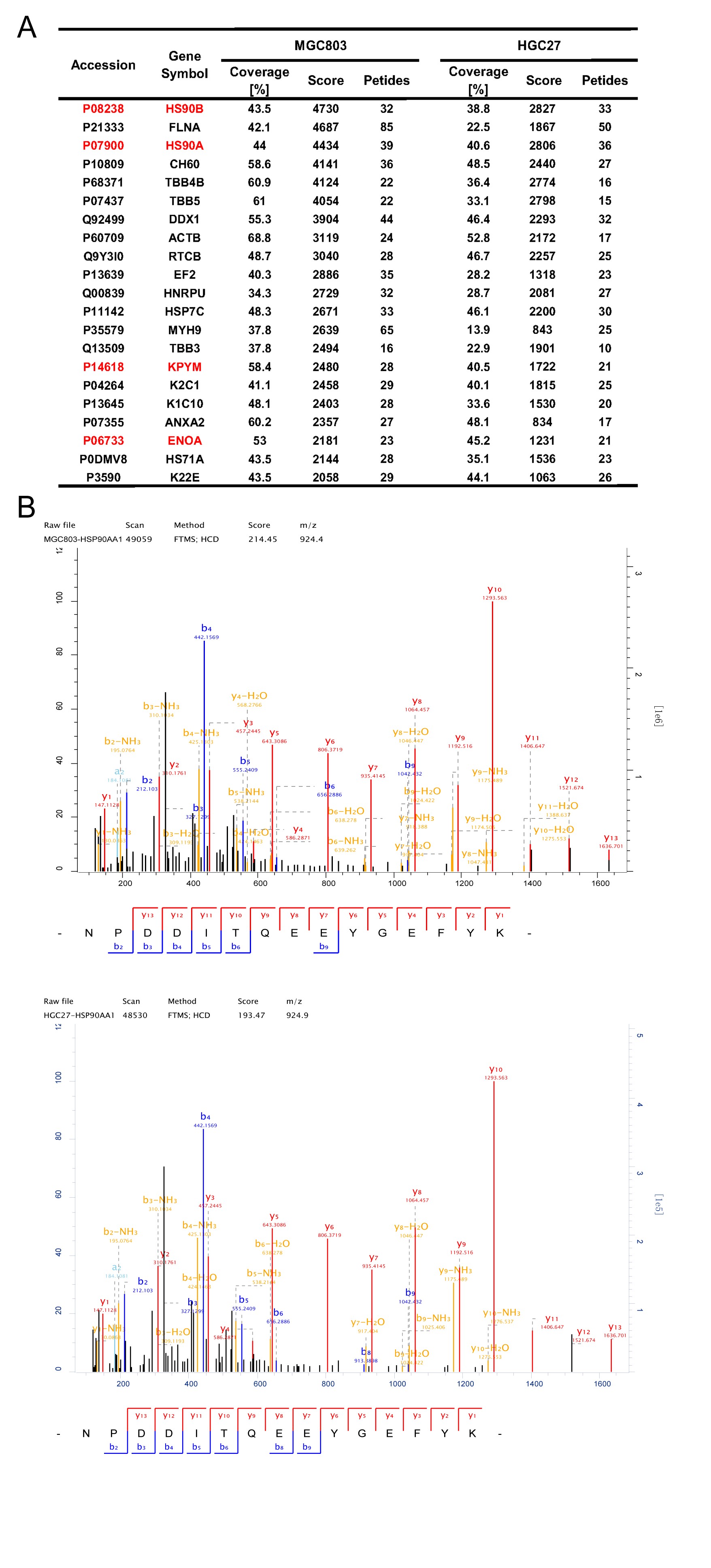


**Figure S2.** Mass spectrometry data analysis of HSP90 pull-down protein

1. Top 20 protein information in the mass spectrometry data of HSP90 pull-down protein in MGC803 and HGC27 cell lines.
2. Secondary mass spectra of HSP90AA1 in MGC803 and HGC27.


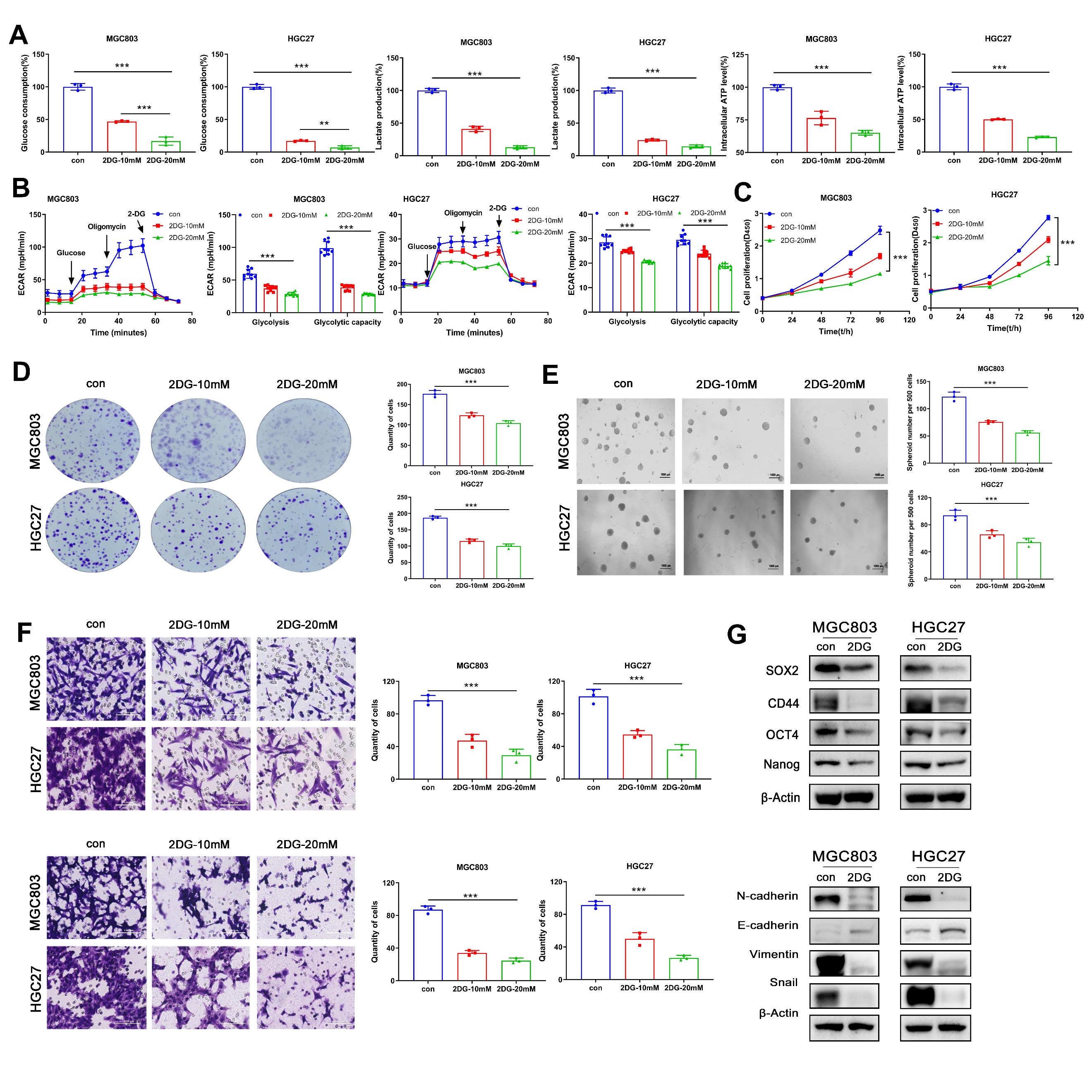
 **Figure S3.** Glycolysis can affect the stem-like characteristics of gastric cancer cells

1. Glucose consumption, lactate production and intracellular ATP production in MGC803 and HGC27 cell were detected after 2-DG inhibition of glycolysis.
2. ECAR were examined in MGC803 and HGC27 cell lines were detected after 2-DG inhibition of glycolysis.
3. CCK-8 show cell proliferation capacity of MGC803 and HGC27 after 2-DG inhibition of glycolysis.
4. The colony-formation ability of MGC803 and HGC27 was detected after 2-DG inhibition of glycolysis.
5. The self-renewal ability of MGC803 and HGC27 was detected after 2-DG inhibition of glycolysis. Scale bar, 1000 μm.
6. The migration and invasion ability of MGC803 and HGC27 was detected after 2-DG inhibition of glycolysis. Scale bar, 100 μm.
7. Expression of stemness markers and EMT-related markers in MGC803 and HGC27 was detected by western blot after 2-DG inhibition of glycolysis.

Error bars indicate mean ± SD. *p < 0.05, **p < 0.01 and ***p < 0.001.


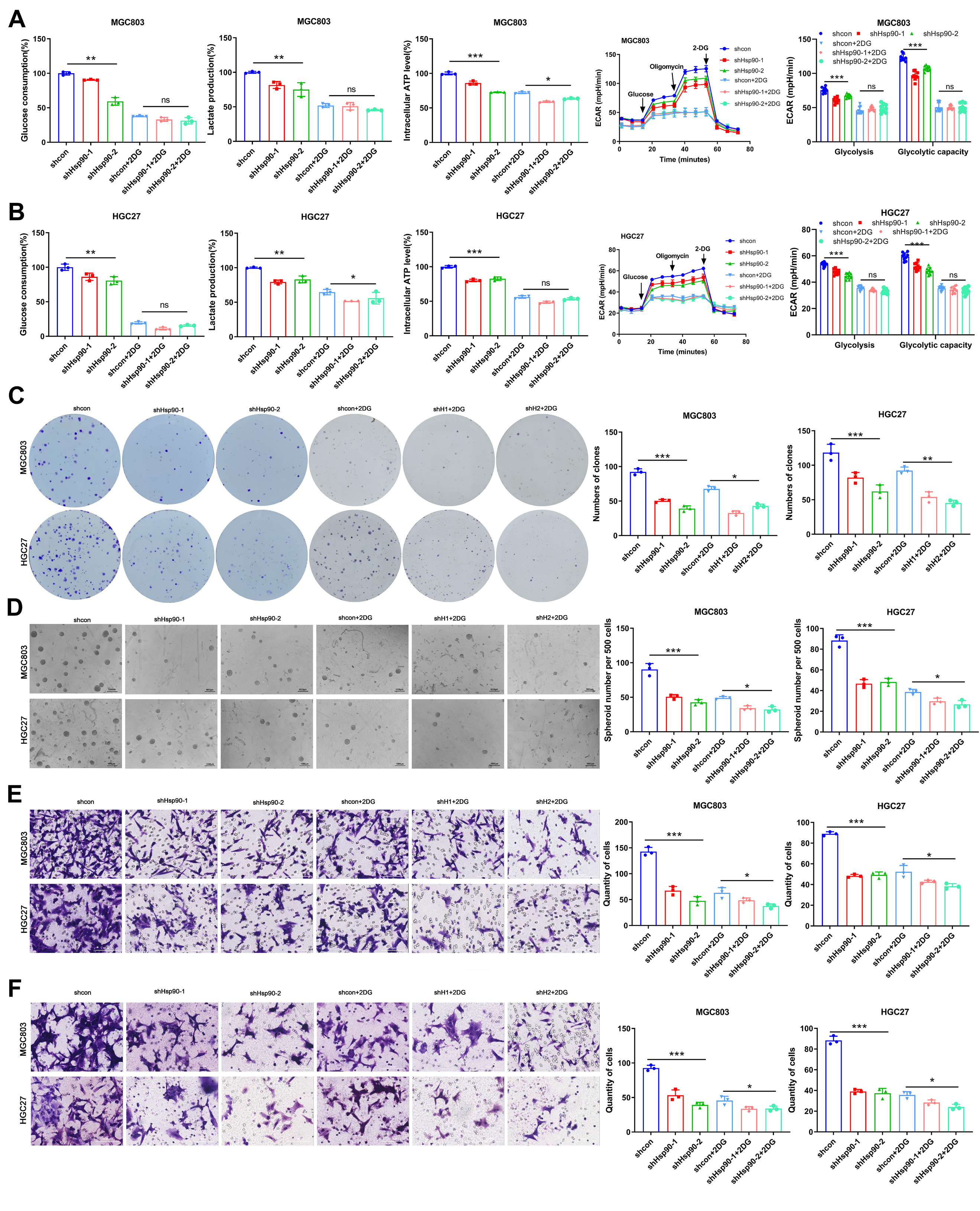


**Figure S4.** The regulation of Hsp90 on gastric cancer depends on glycolysis.

A) Glucose consumption, lactate production and intracellular ATP production in MGC803 and HGC27 stable cell lines were detected before and after 2-DG inhibition of glycolysis.

B) ECAR were examined in MGC803 and HGC27 stable cell lines were detected before and after 2-DG inhibition of glycolysis.

C) The colony-formation ability of stable cell lines was detected before and after 2-DG inhibition of glycolysis.

D) The self-renewal ability of stable cell lines was detected before and after 2-DG inhibition of glycolysis. Scale bar, 1000 µm.

E) The migration ability of stable cell lines was detected before and after 2-DG inhibition of glycolysis.

F) The invasion ability of stable cell lines was detected before and after 2-DG inhibition of glycolysis. Scale bar, 100 µm.

Error bars indicate mean ± SD. *p < 0.05, **p < 0.01 and ***p < 0.001.


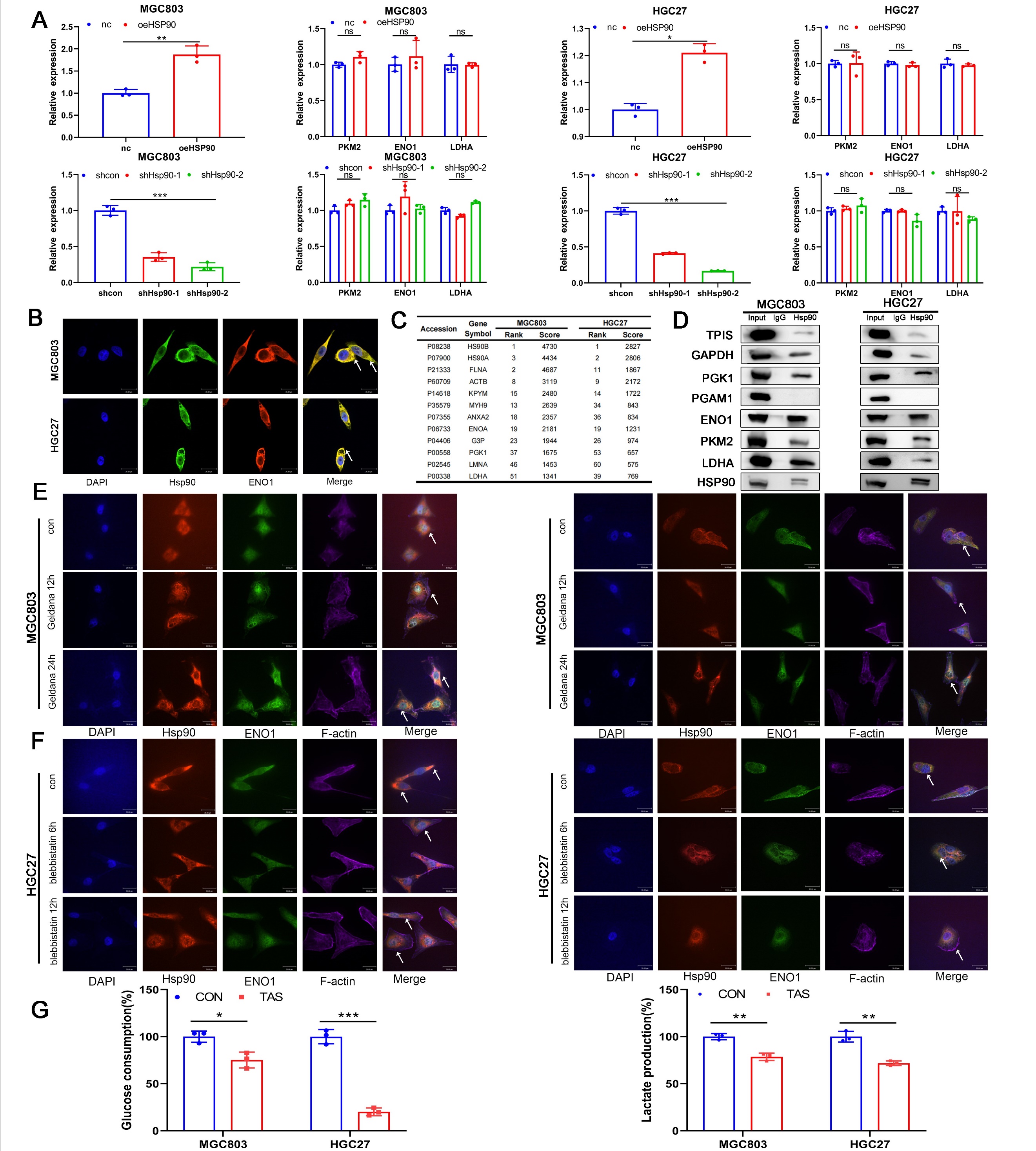


**Figure S5.** HSP90 regulates the regionalized distribution of glycolytic enzyme complexes through cytoskeleton-associated proteins.

1. Expression of glycolytic enzymes in MGC803 and HGC27 stable cell lines was detected by Quantitative RT‐PCR.
2. The co-localization of HSP90 and ENO1 in MGC803 and HGC27 cells was demonstrated by immunofluorescence.
3. Glycolytic enzymes and cytoskeleton-related proteins information in the mass spectrometry data of HSP90 pull-down protein in MGC803 and HGC27 cell lines.
4. MGC803 and HGC27 cells were immunoprecipitated with normal IgG or anti‐Hsp90, and precipitates were analyzed by immunoblotting (IB) with indicated antibodies.
5. The changes of co-localization positions of HSP90 and ENO1 were detected by immunofluorescence after treatment with HSP90 inhibitor GA for 12h and 24h.
6. The changes of co-localization positions of HSP90 and ENO1 were detected by immunofluorescence after treatment with MYH9 inhibitor blebbistatin for 6h and 12h.
7. Glucose consumption and lactate production in MGC803 and HGC27 cell were detected after TAS-116 inhibition of HSP90.

Error bars indicate mean ± SD. *p < 0.05, **p < 0.01 and ***p < 0.001.


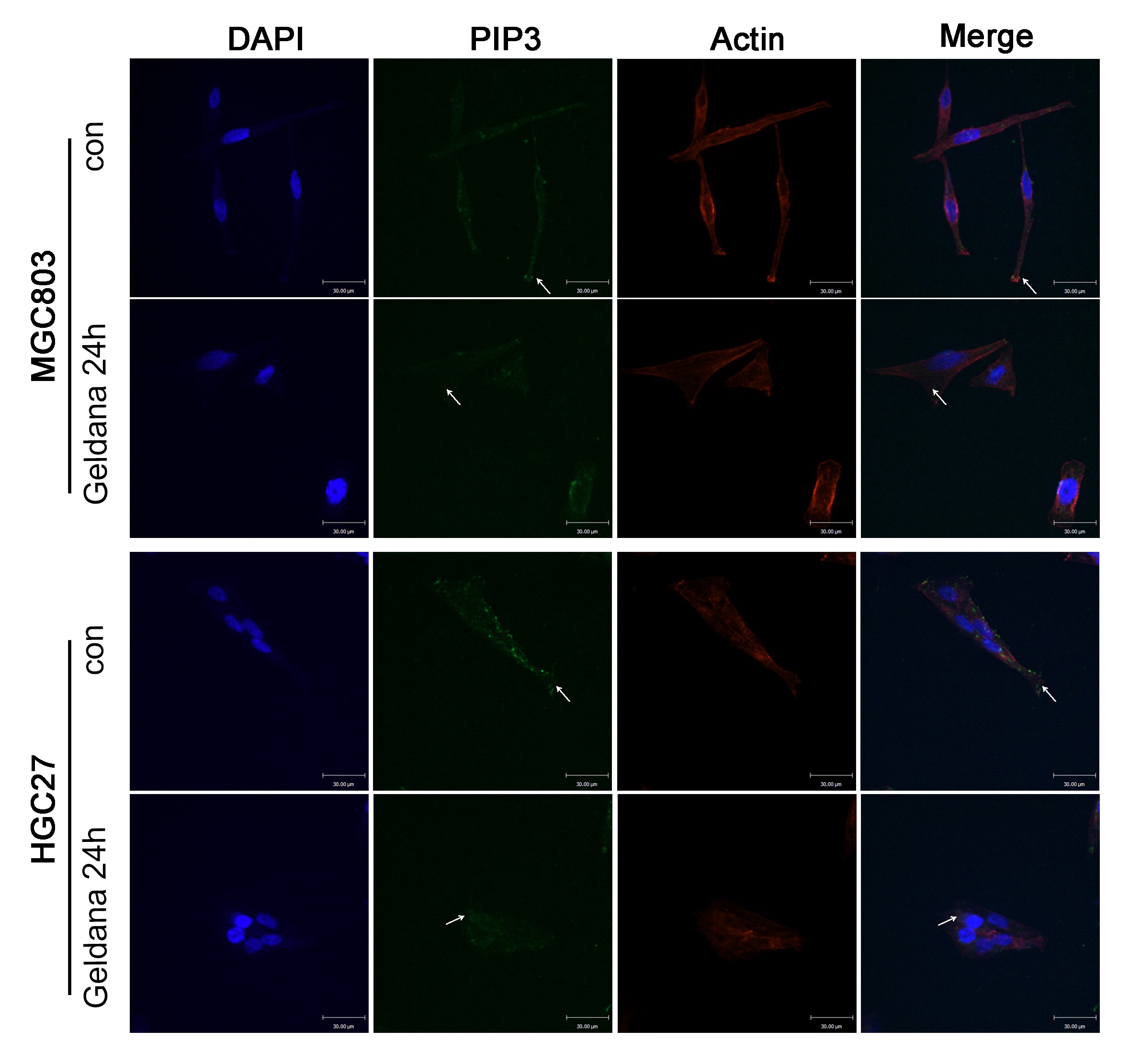


**Figure S6.** HSP90 regulates the generation and the regionalized distribution of phosphatidylinositol (3,4,5)-trisphosphate (PIP3).

**Table S1.** The primer sequences used for qPCR in this study

| Genes | Primer Sequences |
| --- | --- |
| HSP90AA1 | F: CATAACGATGATGAGCAGTACGC |
|  | R: GACCCATAGGTTCACCTGTGT |
| actin | F: CATGTACGTTGCTATCCAGGC |
|  | R: CTCCTTAATGTCACGCACGAT |
| sox2 | F: AAGACGCTCATGAAGAAGGATAA |
|  | R: ACTGTCCATGCGCTGGTT |
| oct4 | F: AGGTATTCAGCCAAACGACCA |
|  | R: GCACGAGGGTTTCTGCTTTG |
| nanog | F: TCTGGACACTGGCTGAATCC |
|  | R: TGACTGGATGGGCATCATGG |
| PKM | F: ATAACGCCTACATGGAAAAGTGT |
|  | R: TAAGCCCATCATCCACGTAGA |
| ENO1 | F: AAAGCTGGTGCCGTTGAGAA |
|  | R: GGTTGTGGTAAACCTCTGCTC |
| LDHA | F: ATGGCAACTCTAAAGGATCAGC |
|  | R: CCAACCCCAACAACTGTAATCT |
